# Advances in Prostate Cancer Biomarkers and Probes

**DOI:** 10.34133/cbsystems.0129

**Published:** 2024-06-27

**Authors:** Keyi Li, Qiao Wang, Xiaoying Tang, Ozioma Udochukwu Akakuru, Ruobing Li, Yan Wang, Renran Zhang, Zhenqi Jiang, Zhuo Yang

**Affiliations:** ^1^ Department of Endoscope, General Hospital of Northern Theater Command, Shenyang, Liaoning, P. R. China.; ^2^ School of Medical Technology, Beijing Institute of Technology, Beijing, P. R. China.; ^3^ Department of Chemical and Petroleum Engineering, Schulich School of Engineering, University of Calgary, Alberta T2N 1N4, Canada.

## Abstract

Prostate cancer is one of the most prevalent malignant tumors in men worldwide, and early diagnosis is essential to improve patient survival. This review provides a comprehensive discussion of recent advances in prostate cancer biomarkers, including molecular, cellular, and exosomal biomarkers. The potential of various biomarkers such as gene fusions (TMPRSS2-ERG), noncoding RNAs (SNHG12), proteins (PSA, PSMA, AR), and circulating tumor cells (CTCs) in the diagnosis, prognosis, and targeted therapies of prostate cancer is emphasized. In addition, this review systematically explores how multi-omics data and artificial intelligence technologies can be used for biomarker discovery and personalized medicine applications. In addition, this review provides insights into the development of specific probes, including fluorescent, electrochemical, and radionuclide probes, for sensitive and accurate detection of prostate cancer biomarkers. In conclusion, this review provides a comprehensive overview of the status and future directions of prostate cancer biomarker research, emphasizing the potential for precision diagnosis and targeted therapy.

## Introduction

Prostate cancer (PCa) is the fifth leading cause of cancer death worldwide [1]. PCa was presented as the second most common cancer among the male population globally, accounting for 14.1% of all incident cancer cases and 6.8% of all deaths in men in 2020 [2]. The prevalence is higher on elderly people, which is escalated by aging population in many countries. Early diagnosis of PCa is essential to improve the survival rate of patients. However, the occurrence and development of PCa is a complex biological process, and it is often challenging to meet the clinical needs only by routine rectal digital examination and PSA level test. Therefore, it is crucial to develop new diagnostic and prognostic indicators that can accurately identify PCa in patients to assess the risk of disease progression and to provide a basis for formulating individualized treatment plans.

Given the limitation of PSA screening, scholars have carried out related research on various forms of combined PSA detection, such as free PSA (fPSA), p2PSA, 4K, and PHI, to improve the differential diagnosis effect of patients with PSA gray area [3]. With the rapid growth of omics detection in recent years, especially the wide application of genomics and proteomics technology, progress has been made in studying PCa’s occurrence and development mechanism. Notably, many genes have been discovered that are connected with the occurrence and progression of PCa, which provides resources for finding new diagnostic biomarkers. TMPRSS2-ERG (transmembrane protease serine 2-ETS transcription factor) gene fusion event is a high-frequency specific rearrangement of PCa, which can be used as a diagnostic index [4]. PCA3 gene is highly overexpressed explicitly in PCa tissues, and its detection has been commercialized. In addition, some microRNAs (miRNAs) in body fluids, such as miR-141, also show the diagnostic value of PCa [5]. Simultaneously, a series of differential proteins in body fluids or tissues of patients with PCa can be detected by mass spectrometry (MS), and these proteins are worthy of attention as potential diagnostic biomarkers. Representative proteins include prostate-specific membrane antigen (PSMA) and AMACR 4. The clinical application of these protein biomarkers needs to be further verified.

**Fig. 1.**
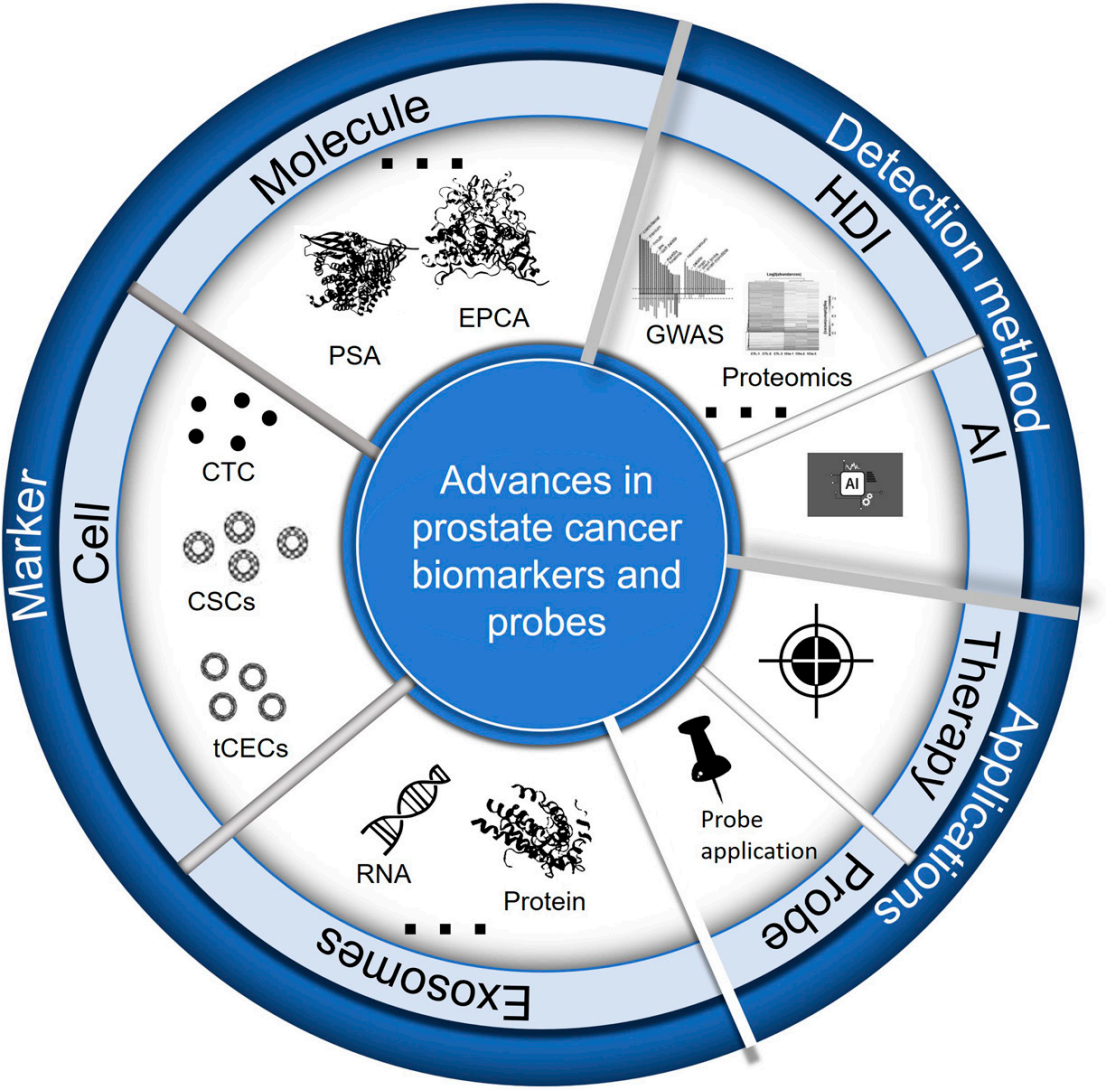
Table of Contents (TOC) diagram of the synthesis.

The aim of this review is to provide a comprehensive overview of recent advances in PCa biomarkers and probes and their applications in targeted therapy and diagnosis. Various types of biomarkers are specifically covered, including molecular biomarkers (genes, noncoding RNAs, and proteins), cellular biomarkers [circulating tumor cells (CTCs), cancer stem cells (CSCs), and circulating endothelial cells (tCECs)], and exosomal biomarkers (RNAs, proteins, DNAs, and lipids). In addition, the review delves into the importance of major advances in biomarker detection by histologic data integration and artificial intelligence analysis to unravel the complex molecular mechanisms underlying PCa development and progression (Fig. 1).

## PCa Biomarkers

### Molecule

PCa is a complex disease, involving various molecular aberrations and cell changes [6]. With the development of biotechnology and genomics, more and more molecular biomarkers play a key role in the diagnosis and treatment of PCa. These biomarkers contain a wide range of biomolecules, including noncoding RNA, genes, and protein, each of which is helpful for understanding the disease mechanism and can be used as a potential target for diagnosis, prognosis, or treatment. Table 1 summarizes the molecular-based biomarkers that show good potential in PCa research.

**Table 1.** Summary of molecule PCa biomarkers and function

PCa biomarkers	Function
UBASH3B	A gene involved in regulating various biological processes, including cellular signaling and immune regulation. This gene’s mRNA and protein expression is potentially related to PCa.
The TMPRSS2-ERG gene rearrangement	The fusion of TMPRSS2 (transmembrane protease serine 2) and ERG (ETS transcription factor) genes, resulting in the overexpression of ERG. ERG overexpression is closely associated with cancer development.
BRCA	A vital DNA damage repair gene in the human body. Mutations in BCRA genes are associated with an increased risk of cancers such as PCa.
SNHG12	An lncRNA, which plays a role in PCa progression and acts as a prognostic biomarker.
Androgen receptor (AR)	A transcription factor activated by androgens, which contributes to control the balance between cell proliferation and cell differentiation, and a transcription factor activated by androgens, which helps to control the balance between cell proliferation and cell differentiation.
PSA	A specific glycoprotein produced by prostate alveolar and ductal epithelial cells. PSA levels are usually elevated in patients with PCa.
PSMA	A membrane surface glycoprotein that is expressed at low levels in normal prostate tissues but highly expressed in PCa tissues.
EPCA	A cytosolic protein encoded by a gene consisting of eight different exons. EPCA-2 is a highly specific EPCA isoform, which is highly expressed in PCa tissues, but lowly expressed in normal tissues.
IKKα	A protein kinase belonging to the IKK family, which plays an essential role in cellular signaling, participating in regulating processes such as inflammation, immunity, and growth of cells, and has a good prognostic value but cannot be used as an independent prognostic biomarker for PCa.
ZIC2	A protein-coding gene essential in embryonic development and tissue differentiation. It belongs to the zinc finger transcription factor family, which is crucial in regulating gene expression and cell fate. Deleting ZIC2 protein expression was associated with poor prognosis.
B7-H4	A protein involved in immune regulation, which belongs to the B7 family of immune checkpoint molecules, and is a potential prognostic biomarker in PCa.
Cytokine (CK)	A class of small molecule proteins produced by cells that transmit signals between cells and regulate biological processes such as immune response, inflammatory response, and cell multiplication.
RAMP1	A membrane protein involved in regulating receptor activity. Signal transduction is highly expressed in PCa tissues, with high specificity.

#### Nucleic acid

(1) UBASH3B

In addition to genes related to amino acid metabolism, genes responsible for regulating T cell receptor signaling have also been found to be candidate prognostic biomarkers for PCa. UBASH3B is a gene involved in the regulation of various biological processes, including cell signaling and immune regulation. Wang et al. [7] detected the mRNA and protein expression of UBASH3B in patients with PCa and benign prostatic hyperplasia (BPH) by real-time fluorescence quantitative polymerase chain reaction and immunohistochemistry and analyzed the gene expression data. The analysis found that this gene’s mRNA and protein expression was elevated in PCa. In contrast, Kaplan–Meier analysis showed that the 5-year overall survival of PCa patients with high UBASH3B expression levels was significantly shorter than that of patients with low UBASH3B levels, which may be a potential prognostic biomarker and correlated with tumor-infiltrating immune cells in the tumor microenvironment [7].

(2) The TMPRSS2-ERG gene rearrangement

The TMPRSS2-ERG gene rearrangement is a common PCa-specific gene rearrangement that involves the fusion of TMPRSS2 and ERG genes, resulting in the overexpression of ERG [8]. In 2005, Petrovics et al. [9] first reported this fusion phenomenon in PCa. Subsequently, Tomlins et al. [10] elucidated the mechanism behind this fusion, revealing the fusion of TMPRSS2 and ERG. TMPRSS2, under the influence of androgens, promotes ERG expression, leading to ERG oncoprotein overexpression in PCa. Numerous studies have verified that abnormal ERG expression, in conjunction with PTEN loss or other molecular alterations, collectively contributes to PCa occurrence and metastasis [11]. According to TCGA data, TMPRSS2-ERG fusion serves as a primary molecular classification factor for localized PCa and a potential prognostic indicator [11].

Furthermore, research indicates that TMPRSS2-ERG gene fusion is present in the majority of metastatic PCa cases, with positive tumors exhibiting a higher propensity for metastasis [12]. It has been shown that ERG expression triggers precancerous cells to accumulate additional mutations by inhibiting oncogene-induced senescence and that gene rearrangements can be used as specific biomarkers of PCa for the diagnosis of PCa [13].

(3) BRCA (BRCA1/BRCA2)

In 2001, Mary Claire King and her team made a groundbreaking discovery by identifying the BRCA1 gene, linking it to early-onset breast cancer, and successfully mapping it to chromosome 17. Subsequently, they located and cloned the BRCA2 gene on chromosome 13, further advancing breast cancer research [14]. Concurrently, research revealed the association of the ESRP1 gene with early invasive PCa. Despite its location on chromosome 8, distinct from the MYC oncogene, ESRP1 plays a unique role in cancer development [15]. Furthermore, the transmembrane protein encoded by the KIAA1324 gene exhibits expression in various cancers and correlates with patient prognosis [15]. While the precise biological function of KIAA1324 remains elusive, its potential as a cancer biomarker warrants further investigation and exploration.

Besides the BRCA genes, approximately 22% of advanced PCa patients display defective DNA damage repair (DDR), linked to genes involved in DNA damage repair like BRCA1 and BRCA2 [16,17]. Studies indicate that mutations in BRCA genes elevate the risk of prostate, pancreatic, breast, and ovarian cancers. Clinical trials have identified BRCA2 gene mutations as an independent prognostic factor for PCa patients [17].

(4) SNHG12

Long noncoding RNAs (lncRNAs) are a class of long-stranded noncoding RNAs greater than 200 nucleotides in length and are an important component of noncoding genomes. Several studies have shown that lncRNAs can act as cancer or tumor suppressor genes. Among lncRNAs, studies have shown that the small nuclear kernel host gene 12 (SNHG12) is associated with cancer types such as prostate, breast, and gastric cancers [18]. Changes in the expression of SNHG12 are associated with tumor cell viability, proliferation, and metastasis and can be used as a prognostic cancer biomarker. Cheng et al. [19] deployed bioinformatic methods in their analysis and found that SNHG12 is closely associated with PCa progression. They also investigated the interaction between miR-133b and SNHG12 in PCa progression, and demonstrated that SNHG12 can be used as a prognostic biomarker for PCa. It was shown in the study that SNHG12 acts as an oncogene by sponging the tumor suppressor gene miR-133b to promote PCa tumorigenesis [20,21].

#### Proteins

(1) Androgen receptor

**Fig. 2.**
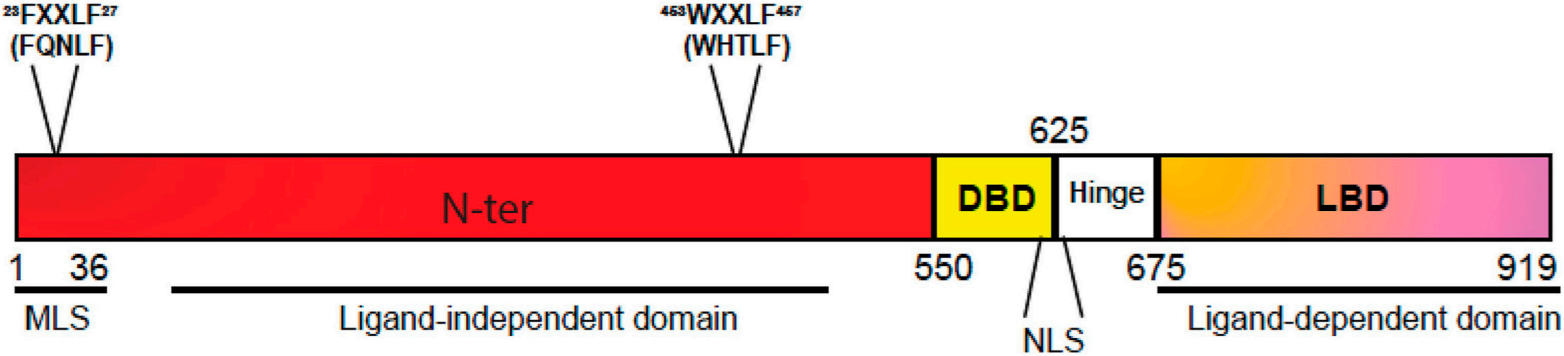
Structure of protein domain of human AR. AR is a protein of 919 amino acids consisting of several functional domains including N-terminal (N-ter) domain, DNA binding domain (DBD), and ligand binding domain (LBD) at the C terminus. The FXXLF motif at amino acids 23 to 27, the WXXLF motif at amino acids 453 to 457, the nuclear localization signal (NLS), and a putative mitochondrial localization signal (MLS) are also depicted [195]. Copyright 2021, *Cancer Letters.*

In 1941, Charles Huggins and Clarence Hodges conducted a groundbreaking study on the relationship between androgens and PCa growth. Their observations revealed that testosterone usage stimulated prostate growth, while castration led to a reduction in the burden of advanced PCa [22]. Subsequent research has elucidated that the androgen effect in PCa is contingent upon the androgen receptor (AR) (Fig. 2), functioning as a hormone-activated transcription factor [23]. Mutations and amplifications of ARs have been documented in both primary PCa and metastatic tumors, with patients undergoing AR antagonist therapy exhibiting a higher likelihood of these mutations. These investigations underscore the significance of AR as a biomarker for PCa, offering crucial insights for further research and treatment in this domain [22].

Since PCa is an androgen-dependent disease, the AR is essential for normal prostate cells. AR helps to control the balance between cell proliferation and cell differentiation, but up-regulation of AR signaling in PCa cells leads to unrestricted accumulation. Androgen deprivation therapy (ADT) is the mainstay of treatment for advanced PCa. However, recent findings indicate that although most advanced PCas respond positively to ADT, many eventually progress to androgen-desensitized PCa. This underscores the continued importance of AR and its downstream signaling pathways in tumor growth [24]. AR aberrations will lead to resistance to ADT and AR-targeted therapies on molecular pathways. Specific abnormalities include AR gene mutations, splice variants, and amplification. These aberrations will increase during tumor progression; therefore, alterations in the AR pathway can be used as prognostic biomarkers for PCa [25].

(2) Prostate-specific antigen

Prostate-specific antigen (PSA) belongs to serine protease family, which is a specific glycoprotein produced by prostatic alveoli and ductal epithelial cells with a molecular weight of 33 kDa. It is currently the most widely used biomarker for PCa screening, diagnosis, risk stratification, and surveillance [26]. Research on antigens characterizing prostate diseases in human semen dates back to the 1970s [27]. The purification of human prostate antigen was first reported in 1979, with its discovery in the serum of patients with advanced PCa following in 1980 [28]. In the same year, enzyme immunoassay was employed to quantify PSA in male serum, initially utilized in 1981 for monitoring PCa patients [29,30]. Originally, PSA testing was intended for monitoring disease progression and treatment response rather than screening. In 1991, it was reported as a screening tool for PCa [31]. It has been discovered that PSA levels are usually elevated in patients with PCa and are initially considered positive when the concentration is higher than 4.0 ng/ml [32]. PSA can be considered a clear indicator of pathogenesis when its concentration in the blood is greater than 10 ng/ml [33]. Recent joint guidelines published by associations such as American Urological Association indicate the corresponding PSA threshold concentrations for PCa prognosis, where men with PSA concentrations of <10 μg/l should be categorized as very low or low risk, men with PSA concentrations of 10 to <20 μg/l should be considered to be at recurrence risk moderate, and men with PSA concentrations of ≥20 μg/l should be considered at high risk for recurrence [34].

However, Jia et al. [35] found that although PSA has a high tissue specificity, its tumor specificity is low, and the PSA test is not exclusive to diagnose the presence of PCa. Its low sensitivity, especially in the gray area, usually leads to overtreatment or missed diagnosis [32]. To improve the specificity and accuracy of the test, ancillary tests are generally performed, including fPSA percentage, PHI, and PCA3 [36]. The findings suggest that the free/total ratio appears to be most clinically useful when PSA reaches levels of 4 to 10 ng/ml, and that detection of the free/total ratio may improve the specificity of surveillance for PCa and reduce the number of patients with negative biopsies [37]. Huang et al. [38] found that adding a fPSA percentage increased the sensitivity range of their test. The Prostate Health Index (PHI) consists of measures of -2proPSA, percent fPSA, and total PSA, and combining the levels of these three proteins resulted in an overall sensitivity of 90% [39]. PCA3 is a noncoding miRNA highly expressed in PCa tissues, while it is barely represented in normal prostate tissues, various other tissues, and organs [40]. The use of combined assays effectively improves the accuracy of PCa diagnosis. It has been pinpointed that the accuracy of PSA detection can be effectively improved using biosensors. In the field of biological information detection and medical treatment, a number of biomarker sensors that are based on electrochemistry, surface plasmon resonance (SPR), nanowires, and other microstructures have been put into commercial application [41].

(3) Prostate-specific membrane antigen

PSMA is a membrane surface glycoprotein expressed at low levels in normal prostate tissues but highly expressed in PCa tissues [42]. Kabay et al. [43] used a disposable and label-free electrochemical immunosensor for the sensitive and selective detection of PSMA to determine whether a patient has PCa. PSMA-PET (positron emission tomography) was useful in customizing treatment to individual patients’ specific needs and assessing response after systemic therapy in patients with advanced PCa [44]. PSMA is mainly used in nuclear medicine in combination with PET imaging to locate prostate tumor cells. The ^68^Ga-PSMA-11 radioactive tracer has been approved by the US Food and Drug Administration (FDA) and European Medicines Agency (EMA) for the study of PCa in different clinical environments [45]. The lesion location displayed by Ga-PSMA PET/CT (computed tomography) is closely related to primary PCa and has high sensitivity and specificity in detecting and locating cancer [46]. PSMA-PET can be used for the diagnosis and localization of PCa, with high predictive value and detection rate for recurrent PCa [47]. In addition to diagnosis and localization, PSMA-PET can also be used for prognostic testing of prostate hyperplasia, with poor detection effect at low PSA levels, while ^18^F-PSMA-1007 performs better in some cases. The combination of PSMA-PET and Fluorodeoxyglucose (FDG)-PET can improve lesion detection rate and predict patient survival more accurately [48]. Therefore, PSMA-PET can be used not only for the diagnosis and localization of PCa but also for predicting patient survival and cancer recurrence rates, with important clinical prognostic value.

(4) Early prostate cancer antigen

Early prostate cancer antigen (EPCA) is a highly relevant biomarker for PCa diagnosis. It is a cytosolic protein encoded by a gene consisting of eight exons. EPCA has been found to be sensitive and specific as a biomarker of PCa [49]. EPCA-2 is an EPCA isoform that is highly specific and highly expressed in PCa tissues but not in normal tissues. Pourmand et al. [50] examined EPCA-2 in 176 patients and performed univariate and multivariate analyses to show that EPCA-2 is a specific biomarker. However, recent studies have shown that the efficiency of single biomarker tests still cannot catch up with combined biomarker tests. Li et al. [49] retrospectively studied several patients with PCa and prostatic hyperplasia to determine EPCA-2 levels. They found that the diagnostic concordance of combined assessment of magnetic resonance imaging (MRI), PSA, soluble E-calmodulin, and EPCA-2 for identifying PCa and BPH was 93%, significantly higher than that of the separate method.

(5) IKKα

Prostate cells are androgen-dependent, and androgen deficiency leads to apoptosis and inflammation. Nuclear factor κB (NF-κB) regulates inflammatory factors. The main component upstream of NF-κB signaling is the IκB kinase (IKK) complex. As a catalytic subunit of the IKK complex, IKKα can activate the IKK complex, further start NF-κB, and allow the activated NF-κB to enter the nucleus, thereby regulating a variety of gene expression, mediating inflammatory responses, immune responses, cellular activity, and other biological processes such as cellular activity. Montes et al. [51] assessed the correlation between IKKα and PCa by evaluating the expression of proteins in cytoplasmic and nucleus by immunohistochemistry in tumor cells. The study revealed that IKKα has an excellent prognostic value but cannot be used as an independent prognostic biomarker for PCa.

(6) ZIC2 protein

ZIC2 is a crucial gene initially identified in human embryos and the nervous system [52]. Studies have revealed that ZIC2 exhibits high expression in PCa tissue, closely correlated with malignant tumor characteristics, including promoting cell migration, invasion, angiogenesis, and tumor initiation, while inhibiting cell apoptosis [53]. ZIC2 is significantly elevated in PCa, nasopharyngeal carcinoma, breast cancer, and acute myeloid leukemia [53,54]. Toth et al. [55] investigated DNA methylation changes between good and poor prognosis PCa cases, used the classification model to predict aggressive behavior in PCa, and found that deletion of ZIC2 protein expression was associated with poor prognosis. These findings strongly support ZIC2 as a biomarker for PCa. At the same time, ZIC2 plays a role as an oncogene and could serve as a biomarker for PCa prognosis.

(7) B7-H4

B7-H4 is normally expressed in antigen-presenting cytosolic T lymphocytes and can inhibit T cell proliferation, cytokine (CK) secretion, and immunotoxicity. B7-H4 is a molecular biomarker associated with tumor progression and prognosis in ovarian, renal cell, pancreatic, hepatocellular, and gastric cancer patients. It has been analyzed as a potential prognostic biomarker in PCa by a bioassay [56].

(8) Cytokine

**Fig. 3.**
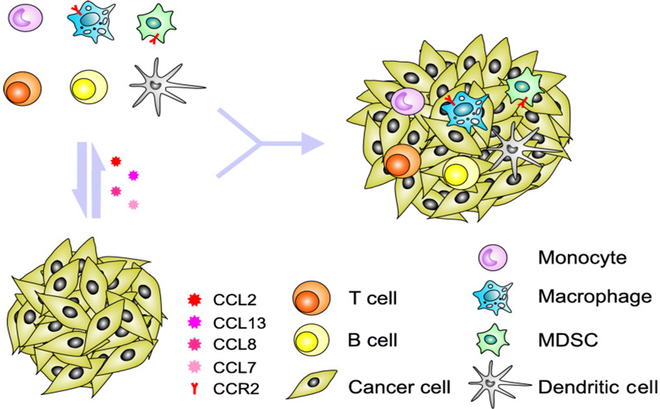
Tumor cells produce chemokines that drive the production of various regulatory immune cells, followed by the production of CKs, chemokines, and other molecules that promote the immune escape of tumor cells [58]. Copyright 2020, *Cell Commun. Signal.*

CKs are a class of small molecule proteins produced by cells that transmit signals between cells and regulate biological processes such as immune response, inflammatory response, and cell proliferation. CKs play an important role in the diagnosis of PCa and have been extensively studied as biomarkers associated with the disease (Fig. 3). Interleukin-6 (IL-6) [57] is an inflammation-associated CK with a high expression level associated with PCa aggressiveness, tumor growth, and poor prognosis. In addition, dysregulation of chemokine expression is closely associated with PCa, and it has been found that up-regulation of CCL2/CCR2 and various immune conditions in PCa are associated with cancer progression, metastasis, and recurrence [58]. It is also found that obesity is closely related to CKs and PCa [59]. The variety of CKs associated with PCa is diverse. Gong et al. [58] used a bioinformatics approach to validate 21 up-regulated and 17 down-regulated CKs in PCa, which are valid diagnostic biomarkers for the disease.

(9) Receptor activity modifying protein 1

Receptor activity modifying protein 1 (RAMP1) is a membrane protein that regulates receptor activity and signaling and is a biomarker associated with PCa. RAMP1 is expressed at high levels in PCa tissues, and its specificity is high [60]. Consequently, detecting the level of RAMP1 in prostate tissue or serum serves as a diagnostic indicator of PCa.

(10) Sialylated glycoprotein

Sialylated glycoproteins are a class of glycoprotein molecules with salivary acid modifications, which are widely found on cell surfaces and in body fluids and play essential roles in various biological processes. It has been found that the expression levels of sialylated glycoproteins are usually higher in PCa tissues [57]. Not only that, some studies have found that targeting Siglec–sialic acid interactions is helpful in treating PCa [61]. Therefore, PCa diagnosis can be made by detecting sialylated glycoprotein expression in tissue samples or by circulating sialylated glycoprotein levels in body fluids such as blood or urine.

### Cell

In addition to molecular biomarkers, PCa research has increasingly focused on cellular biomarkers, which provide valuable insights into the pathogenesis and progression of the disease [62]. Cellular biomarkers encompass various cell types and populations that exhibit distinctive characteristics or behaviors in the context of PCa [62]. Table 2 summarizes several key cellular biomarkers. These biomarkers include CTCs, CSCs, and tCECs, each offering unique opportunities for noninvasive detection, monitoring of disease progression, and identification of therapeutic targets. The study of cellular biomarkers has emerged as a promising frontier in PCa research, complementing molecular biomarker discoveries and facilitating a more comprehensive understanding of this complex malignancy.

**Table 2.** Summary of cellular PCa biomarkers and function

PCa biomarkers	Function
Circulating tumor cell (CTC)	A cancer cell that detaches from the primary tumor and enters the bloodstream, which can be detected in the blood and bone marrow of PCa patients.
Cancer stem cells (CSCs)	CSCs are defined as cells that have self-renewal within cancer tissue. In PCa, CD144 and CD133 are highly expressed.
Circulating endothelial cells (tCECs)	tCECs have been identified as a novel blood biomarker for cancer with clinical significance, with a negative detection rate of over 90% in PCa testing.

#### Circulating tumor cells

**Fig. 4.**
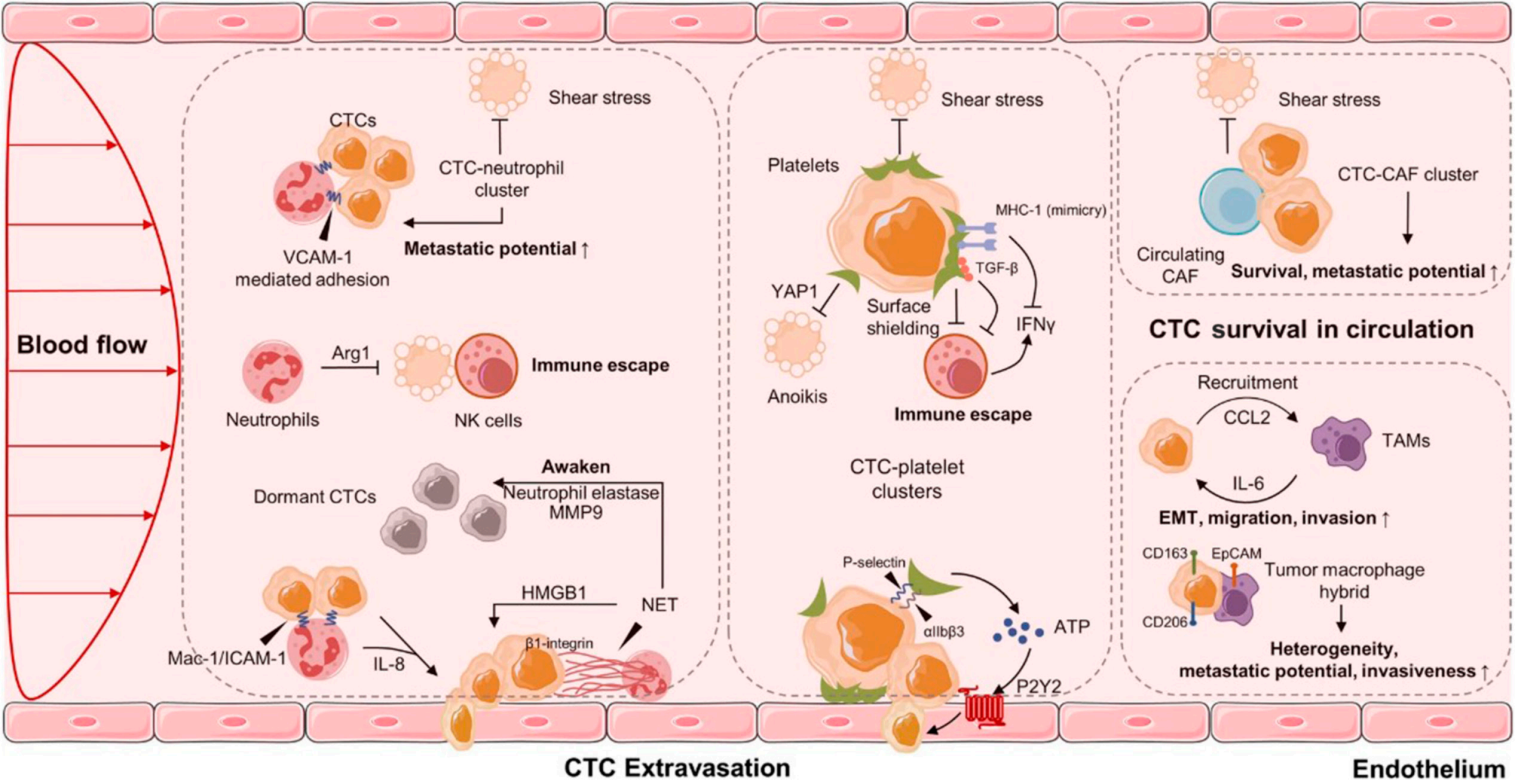
CTCs in the blood microenvironment and their interaction with neutrophils, platelets, CAFs, and TAMs. [63]. Copyright 2021, *Sig Transduct Target Ther.*

Tumor-associated cells in the blood microenvironment interact with neutrophils, platelets, cancer-associated fibroblast (CAFs), and tumor-associated macrophages (TAMs), being excepted to be biomarkers for PCa [63] (Fig. 4). Tumor-associated cells are also expected to be biomarkers for PCa. Tian et al. [64] noted that CTC positivity is an adverse prognostic biomarker for early progression, and CellSearch, an FDA-approved platform for predictive use in PCa based on the detection and enumeration of CTCs using immunomagnetic capturing and fluorescence imaging, demonstrated the results of CTCs as a predictive biomarker for PCa. Meanwhile, many studies have shown that relevant tumor cells can be detected in PCa patients’ blood and bone marrow. With the advent of next-generation sequencing (NGS) and sensitive CTC assay analysis in the plasma of cancer patients, CTCs can be a beneficial liquid biopsy prognostic tool [24]. Before cancer detection and analysis, CTCs need to be enriched before they can be used in subsequent research. Xu et al. [65] proposed a three-dimensional (3D) stacked multi-stage inertial microfluidic sorting chip for high-throughput enrichment and convenient downstream analysis of CTCs. The system can be easily integrated with mobile sample detection methods to realize rapid CTC analysis and help the clinical diagnosis of cancer.

#### Cancer stem cells

**Fig. 5.**
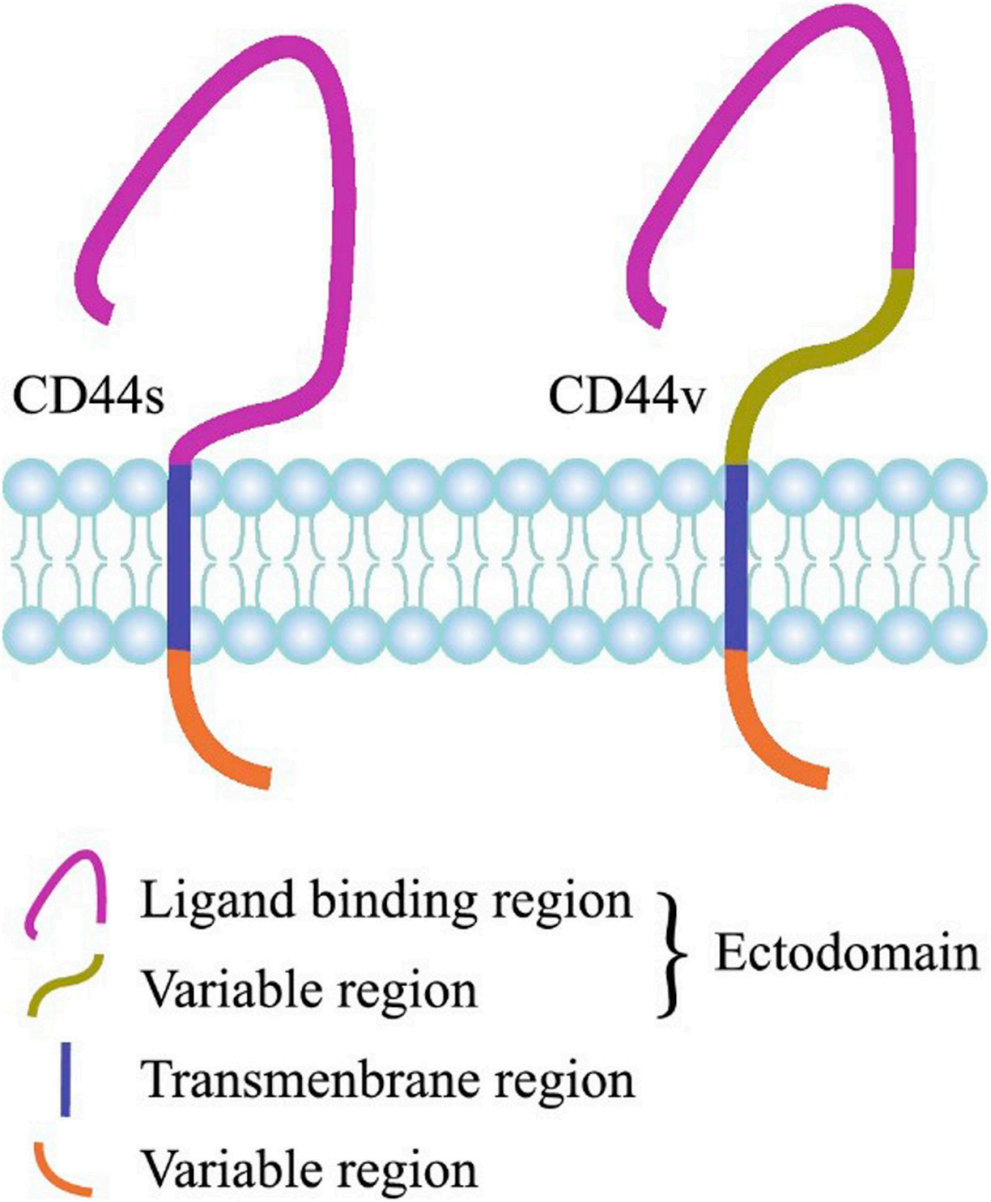
Structure of CD44 protein. CD44 mainly consists of three regions, including extracellular domain, transmembrane domain, and intracellular domain. Compared to CD44s, the extracellular region of CD44v protein additionally contains a variable domain [196]. Copyright 2020, *Exp Hematol Oncol*.

CSCs are defined as cells with self-renewal and proliferation abilities within cancer tissue [66]. Research has shown that CSCs may play an important role in resistance to traditional cancer therapies, and investigating CSCs may help discover new therapies for PCa [67]. PCa stem cell biomarkers encompass multiple key molecules, including integrins, CD44, CD133, CD166, and CD117 [67]. In PCa, integrins are often overexpressed. Specifically, α2-integrin and EZH2 are expressed in a small fraction of cancer cells, indicating their potential as stem cell biomarkers [68–70]. CD44 is a single-channel type I transmembrane protein associated with extracellular matrix signaling (Fig. 5). In PCa, CD44-positive cells exhibit stem cell characteristics and express high levels of stem genes [71,72]. CD133 is a glycoprotein widely expressed in various stem cells and endothelial progenitor cells. In PCa, CD133 expression levels are associated with tumor grading and prognosis [73–76]. CD133^+^ basal cells may be the cells of origin for PCa [77]. CD166 belongs to the immunoglobulin (Ig) family of type I transmembrane proteins, mediates intercellular interactions, and has been used as a prognostic marker for various cancers [78]. CD117 (also known as c-Kit) is a receptor tyrosine kinase protein that has been used as an important cell surface marker for recognizing hematopoietic progenitor cells in the bone marrow and is associated with the ability of cells to self-renew and cancer progression [79–81].

#### Circulating endothelial cells

tCECs have been identified as a novel blood-based biomarker for clinically significant cancer [82]. Research indicates that tCECs can serve as biomarkers for PCa [83]. Studies have demonstrated that the negative predictive value (NPV) of tCEC screening for PCa exceeds 90%, allowing for the safe exclusion of over 70% of negative prostate biopsies. Furthermore, there exists a negative correlation between tCECs and the overdiagnosis of clinically insignificant cancers, potentially reducing overdiagnosis by 40% [83]. While tCECs show promise as a potential biomarker for PCa, further research is necessary to confirm their clinical effectiveness and accuracy in diagnosis and screening.

### Exosomes

**Fig. 6.**
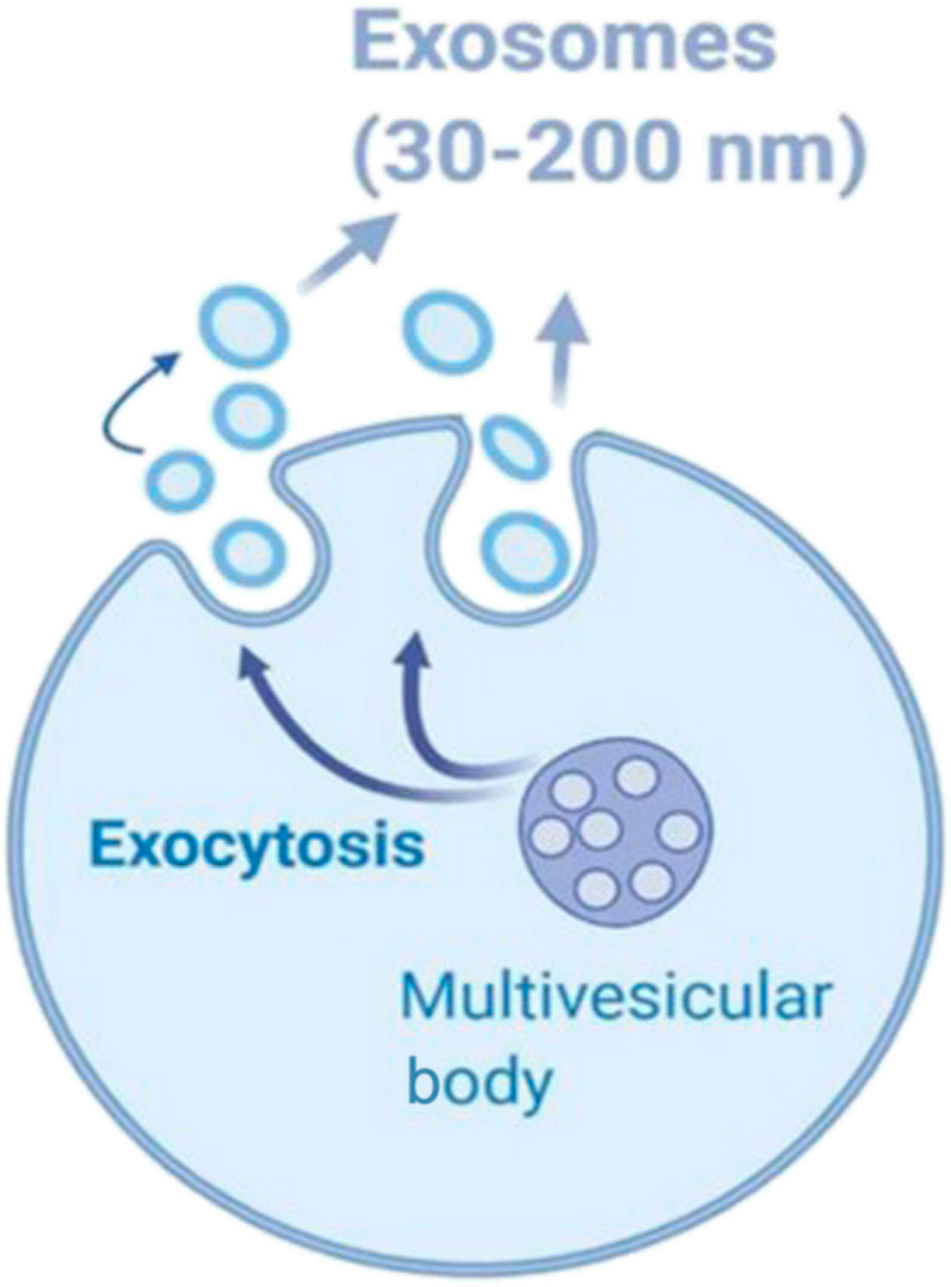
Schematic diagram of EV secretion. EVs are produced by the multivesicular body and released by exocytosis, and they are spherical with a diameter ranging from 30 to 200 nm in diameter [84]. Copyright 2021, *Cell Commun Signal.*

EVs are cellular particles secreted and released outside the cell and encapsulated by a lipid bilayer. EVs are produced by endocytosis and released by exocytosis, and they are spherical with a diameter ranging from 30 to 200 nm [84] (Fig. 6). EVs mainly contain DNA, RNA, proteins, and lipids. In recent years, it has been found that EVs play an essential role in transmitting information between prostate cells, and lesions related to the prostate gland may be manifested by changes in the content or structure of EVs. After EVs are released into the intercellular space through the cytosol, they can mediate intercellular communication between tumor and stromal cells, especially between CAF [85]. EVs can serve as biomarkers of PCa in cancer detection, cancer metastasis control, and prognostic testing.

#### RNA

EVs contain various RNAs closely related to the occurrence and metastasis of PCa, and EV-RNAs can be utilized for the initial diagnosis of PCa. Liquid biopsy refers to a method of tumor diagnosis and monitoring by analyzing tumor-associated molecules in biological fluids such as blood and urine, and several studies have identified specific exosomal miRNAs from blood and urine [86]. The up- and down-regulation of their types and levels are expected to serve as specific biomarkers for liquid biopsy of PCa [87]. The variety of miR-21 and miR-375 from urinary EVs can better differentiate between prostate patients and healthy individuals [88]. Among many exocrine RNA, two miRNAs, mir-26b-5p and miR-98-5p, have higher accuracy in determining PCa in patients. In a recent study, the level of miR-4732-3p was shown to reflect the grade classification of PCa, and the prediction results demonstrated that the accuracy of miRNAs for the judgment of PCa was higher than that of the most commonly used PSA indicator [89]. This shows that EV-RNA can be used for the diagnosis of PCa. RNAs contained in EVs are associated with metastasis of PCa cells. They can play an important role in signaling and protein translation on the surface of cancer cells, and this role is more pronounced in bone metastasis [90]. In the EVs secreted by metastatic PCa cells, the levels of two miRNAs, miR-141 and miR-13, are increased, and they enhance epithelial–mesenchymal transition (EMT), which can promote the metastasis of PCa cells [90,91]. EV-RNAs play an important role in the metastasis of tumor cells, and they can be applied as biomarkers for metastatic PCa. It can be applied as a biomarker in the examination and prediction of diagnosis and prognosis, and targeted therapy against EV-RNA has also become one of the potential preventive therapies for metastatic PCa.

#### Protein

The EVs of prostate cells contain many different types of proteins. In prostate lesions (e.g., PCa), the types and amounts of proteins in their EVs change considerably. Some of the EVs secreted by prostate cells enter the urine, and a variety of proteins in the urine EV are thought to be specific for PCa [92]. Abnormally elevated levels of one of these proteins, FABP5 (a fatty acid binding protein), have been associated with developing PCa [93]. Several studies have indicated that proteins in urine EV can be utilized as PCa-specific biomarkers in the diagnostic testing of PCa [92,94]. This test requires only taking the patient’s urine for analysis without the need for puncture biopsy of the prostate site, which provides better clinical results. Unlike urine, abnormally elevated levels of ERG proteins contained in serum EV are associated with advanced PCa, risk of cancer, and recurrence. Multiple proteins of serum EV may improve diagnostic procedures for PCa and provide information for prognostic PCa testing. The analysis of serum EV proteins has been widely used in other cancer types [95], and this assay also has high clinical value in PCa.

#### DNA

Some studies have shown that EV DNA components also play an essential role in the diagnosis and prognosis of PCa. Large extracellular vesicles (L-EVs) isolated from the plasma of PCa patients are chromosomal DNA-rich EV populations that include large segments up to 20,000 base pairs (bp) long. They contain genes frequently mutated in metastatic PCa cancer cells (MYC, AKT1, PTK2, KLF10, and PTEN), and the amount of DNA in L-EVs is positively correlated with the development of PCa [96]. This finding points to the potential value of exosomal L-EVs as diagnostic biomarkers for PCa. Analysis of plasma L-EV’s DNA, such as gene sequencing, can help determine the presence of PCa in patients. In a 2018 report evaluating the concentration of circulating free DNA (cfDNA) fragments in patients’ plasma after paclitaxel chemotherapy (a PCa treatment), it was found that cfDNA was abnormally elevated in PCa patients before treatment and that as paclitaxel-based treatment was initiated, cfDNA concentrations decreased significantly in patients who benefited from paclitaxel chemotherapy [97]. cfDNA comprises short DNA fragments (<200 bp) shed into the circulation from apoptosis or necrosis of normal and tumor cells. This indicates that the concentration of cfDNA can indicate the presence or absence of PCa, which can be used as a biomarker in the diagnosis and treatment process to guide the provision of improved and more precise diagnostic and treatment tools.

#### Lipids

Most of the studies analyzed characterized the biomarker role of RNA, proteins, and DNA in EVs for PCa, and there are fewer studies about lipids in EVs. Cholesteryl ester (CE) is a lipid-like substance formed by cholesterol and fat. In PCa, the amount of CE in EVs correlates with tumor progression and metastasis and can differentiate between PCa and BPH [97]. Changes in lipids in the EV can provide important information related to the advancement and prognosis of PCa. It should be noted that this information is not firmly oriented and needs to be tested in combination with other biomarkers.

## Methods Applied to the Discovery of New PCa Biomarkers

### Histology data integration

Histomics is a high-throughput research method in the field of systems biology, through a large amount of data collection and analysis, including genomics, transcriptomics, proteomics, metabolomics, as well as the interaction network and functional genomics at multiple levels [98]. With the emergence of NGS tools and the continuous development of MS, researchers can achieve high-throughput sequencing and in-depth analysis of protein expression, modification, and metabolites at the level of individual cells, and genomics technologies provide emerging means of analyzing the molecular characteristics and biological mechanisms of diseases [99,100]. In recent years, large-scale genomics studies have been conducted for different PCa stages [101]. Histomics data integration is the process of integrating and analyzing histomics data from many different levels. This provides a more comprehensive view of PCa disease by bringing these data together to discover correlations between different histological levels, and researchers can gain a more comprehensive understanding of the biological characteristics of PCa [102].

#### Genome-wide association study

Genome-wide association study (GWAS) is an important research method in genomics, which is a research method to search for genetic factors associated with complex diseases by genome-wide typing of large-scale population DNA samples with high-density genetic markers [e.g., single-nucleotide polymorphisms (SNPs) or copy number variations (CNVs)]. In recent years, GWAS has been successful in resolving the genetic components of many complex human diseases, including PCa [103], and a large number of studies have analyzed the genetic variant loci of PCa-associated cells through GWAS data to reveal the risk gene regions for pathogenesis, which can be used as a biomarker to provide important value for the study of the pathogenesis of PCa and clinical application. SNP is a change in a single nucleotide in the DNA sequence of a gene, and it is one of the most common types of genetic variants. Many studies have shown that more than 100 SNPs are associated with the risk of PCa, and the SNP–SNP interaction network plays an important role in the diagnosis and treatment of PCa [104,105]. Hua et al. [106] showed that the PCa high-risk SNP rs11672691 chain imbalance of SNP rs887391 activated PCAT19-long by decreasing the binding of transcription factors NKX3.1 and YY1 to the promoter of PCAT19-short, resulting in a weak promoter activity but a strong enhancer activity, followed by activation of PCAT19-long. PCAT19-long interacts with HNRNPAB to activate a group of cell cycle genes associated with PCa progression, thereby promoting PCa tumor growth and metastasis [106]. This finding elucidates the biological mechanism of SNPs and provides the principal support that SNPs can be used as biomarkers for PCa, proving that the search for PCa-related SNPs through genomics is a powerful research tool for discovering new biomarkers. In recent years, several GWAS-based genomics studies have pointed out that several genetic variants in different regional ethnic groups (including West Africa, Japan, and Europe) are associated with PCa susceptibility, but there are some regional ancestry differences in the variant genes [107–109]. To reduce pedigree differences and obtain universal results, Conti et al. [110] analyzed GWAS data combining 107,247 PCa cases and 127,006 controls (including men from European, African, East Asian, and Hispanic populations) and analyzed between 5.6 million and 16.8 million genotypes. Insertion/deletion variants associated with PCa risk were examined, and 86 new independent genetic loci associated with PCa risk were identified, which had a genome-wide association significance threshold of *P* < 5.0 × 10^−8^. This study suggests that taking advantage of big data analysis in genomics can help researchers identify common genetic loci associated with PCa with greater precision in the face of the differences that exist among different ethnic groups in different regions. These genetic loci can play an important role as biomarkers in PCa detection and risk prediction.

#### Transcriptomics

Transcriptomics reveals gene expression levels by measuring and analyzing transcription product RNA. RNA sequencing (RNA-Seq) is a commonly used analytical method for transcriptomics. It provides comprehensive transcriptomic information including gene expression levels, splicing variants, and RNA sequence variants by measuring and analyzing transcription products such as mRNA, miRNA, and other noncoding RNAs in RNA samples. Alkhateeb et al. [111] used a large amount of data provided by RNA-Seq technology to compare normal and malignant PCa datasets and found differences in HEATR5B, DDC, and GABPB1-AS1 gene expression, which can be used as biomarkers for diagnosing PCa. At the same time, it was found that PTGFR, NREP, SCARNA22, DOCK9, FLVCR2, IK2F3, USP13, and CLASP1 genes were differentially expressed between stage II and subsequent stages of the disease, which can be used as potential biomarkers for the prediction of PCa progression [111]. By analyzing a large number of RNA molecules simultaneously through transcriptomic approaches, researchers can identify multiple RNA molecules or gene loci associated with PCa development and progression and analyze possible interactions. Robinson et al. [112] conducted prospective whole-exome and transcriptome sequencing of bone or soft tissue tumor biopsies from 150 patients with metastatic castration resistant prostate cancer (mCRPC). They discovered that compared to primary PCa, mCRPC exhibited significantly increased frequency of abnormalities in BRCA2, BRCA1, and ATM, along with some individual differences in other gene variants. This finding offers new treatment options for patients seeking personalized precision medicine. Another study pointed out that for multifocal prostate, obtaining RNA biomarkers for low-grade cancer foci did not predict high-grade foci in commercial prognostic tests, further deepening the accuracy and utility of transcriptomics for PCa biomarker analysis [113]. Transcriptomics can provide an overall gene expression profile of PCa tissues or cells, allowing researchers to clarify the pattern and level of gene expression in disease development, which can help researchers discover new susceptibility genes or regulatory loci.

#### Proteomics

Proteomics focuses on the composition, structure, function, and interactions of proteins in living organisms and has a tremendous impact on early cancer detection, diagnostic improvement, recurrence prevention, treatment response monitoring, and improved survival outcomes [114]. Electrophoresis and MS analysis can identify protein interactions and analyze the high-level structure of proteins, which is an important tool for proteomics research and analysis. The continuous development of MS technology, such as the improvement of scanning speed and mass resolution, allows targeted proteomics detection to be performed by noninvasive liquid biopsy, expanding the direction for proteomic studies of PCa to discover abnormal proteins or abnormal protein regulatory pathways [115]. The study by Davaliev et al. [116] used 2D difference gel electrophoresis (DIGE) and MS to compare the proteomic analysis of PCa and BPH tissues and demonstrated that nine proteins, including CSNK1A1, ARID5B, LYPLA1, PSMB6, RABEP1, TALDO1, UBE2N, PPP1CB, and SERPINB1, are dysregulated in PCa tissues and can be used as emerging PCa biomarker with a role in diagnosis. Another study used iTRAQ 3D LC MS to analyze over 1000 proteins histologically in high-quality serum samples and found that seven targeted proteins [six potentially interacting, including recognized cancer markers such as tumor necrosis factor (TNF), signal transducer and activator of transcription 3 (STAT3), *NF-κB*, and IL-6] could be present as biomarkers in PCa [117]. However, the amount of tissue samples obtained in disease diagnostic proteomics analyses is small, and certain MS methods do not allow the detection of large numbers of samples. To address this problem, Turiák et al. [118] investigated biopsies in the form of tissue microarray (TMA) to reduce sample volume and improve sensitivity, and also found that PCa proteins vary greatly with cancer grade and identified protein pathways associated with PCa, making a prospective contribution to PCa biomarkers. Taken together, these studies show that proteomics can analyze the expression levels, modification modes, or interactions of PCa tissue proteins at a large-scale level and discover abnormal proteins associated with PCa. Through highly sensitive identification and quantitative determination by chromatography and MS instruments, proteomics has become an important method for studying PCa susceptibility proteins.

#### Metabolomics

Metabolomics uses high-throughput analytical techniques, such as MS and nuclear magnetic resonance (NMR), to study the overall spectrum of metabolites produced by an organism, which can identify changes in metabolites and abnormalities in metabolic pathways in focal areas to reveal metabolic profiles of organisms in different states. In the study of PCa pathology, the study of metabolomic differences between tumor cells and normal cells can help to discover new biomarkers and provide early diagnosis of cancer and prognostic options [119]. Commonly used metabolomics analysis methods mainly include chromatography-MS, high-resolution MS, and NMR techniques. In the study of cancer cell metabolomics, liquid/gas chromatography-MS (LC/GC-MS) has the characteristics of high resolution and high sensitivity and plays an important role in the discovery of biomarkers and exploration of pathological mechanisms. Several studies have identified multiple metabolites that are associated with a high risk of PCa development by comparative analysis of metabolomics in urine or plasma from normal and PCa patients using LC-MS or GC-MS. These metabolites (including urea and amino acids) are associated with circulatory dysregulation in cellular metabolism and can be used as biomarkers for the early prevention and diagnosis of PCa to help identify men at high PCa risk [120–122]. This suggests that metabolomic analysis of PCa tissues using LC-MS or GC-MS can be effective in identifying PCa-susceptible metabolic end products. However, when performing large-scale metabolomics studies, LC-MS is limited in the translation of clinical diagnosis due to the long data acquisition time. Researchers proposed a high-resolution MS method, combined with a segmented flow approach, to analyze urine samples and found that branched-chain amino acids, among others, maybe a novel tool for PCa diagnosis and prognosis [123]. This analytical method, which allows for faster analysis of the sample’s to-be-tested histology and discovery of pathologically altered metabolic molecules while ensuring accuracy, offers greater advantages in terms of clinical applications. In the face of to-be-tested samples that cannot be ionized, MS is difficult to analyze, and NMR technology can play a key role as a new technological tool in metabolomics research. Yang et al. [124] analyzed the urine samples of cancer and noncancer patients using ^1^H NMR and found that guanidinium acetate, phenylacetylglycine, and glycine were potential biomarkers of PCa. Abnormal changes in metabolites, which are end products of cellular reactions, are more closely linked to lesions exhibited by PCa compared to upstream genes. By analyzing metabolomics, researchers can pinpoint metabolic abnormalities associated with PCa, which is important for the discovery of biomarkers for PCa.

#### Integration of histologic data

The pathological mechanism of PCa has complexity and is related to multiple factors such as environment and cells. The analysis of a single histology can help to gain insight into the mechanism of PCa and search for potential biomarkers, but it may overlook the interactions and comprehensive effects between different histological levels. More and more studies have been conducted to analyze the connection between multiple histological data by integrating the histological data for more in-depth and comprehensive analysis and study of PCa, which can provide precise guidance for personalized medicine and precision treatment.

(1) Genomics–transcriptomics

Combined genomics–transcriptomics analysis plays an important role in PCa research. By integrating genomics and transcriptomics data, it is possible to identify potential driver genes for PCa, analyze the transcriptional network of PCa pathogenesis, and explore new biomarkers, which can provide new directions for clinical diagnosis and treatment of PCa. Yun et al. [125] found significant differences in the expression of SPINK1 and SP8 genes as potential biomarkers in the transcriptomic and genomic analyses of samples from patients with different types of PCa at the primary site. By analyzing the transcriptome and genome of samples from different primary sites, they found that there were significant differences in the expression of SPINK1 and SP8 genes, which can be used as potential biomarkers and help to classify PCa patients with a higher risk of metastasis. Meanwhile, another study demonstrated that genetic variants of PCDH9 and PLXNA1 were associated with PCa progression and that overexpression of *PLXNA1* led to further progression of PCa and worse prognosis by analyzing the whole genome and transcriptome sequencing of untreated PCa patients [126]. Ye et al. [127] demonstrated that by investigating PCa DNA, methylation variation (MET), and mRNA data of PCa, established a risk assessment model, integrated the multi-omics data, and elucidated the potential causative genes of PCa to provide new targets for the treatment of PCa patients. In conclusion, genomics pays more attention to the genes that undergo gene mutation in the disease, while transcriptomics pays more attention to the changes in gene expression level and the differences in RNA structure or concentration. Combining the analysis of genomics and transcriptomics helps researchers to analyze the two interrelated perspectives, namely, DNA and RNA; to find out the gene variations related to PCa from a more comprehensive perspective, gene expression level changes, transcriptionally obtained RNA modification abnormalities, or RNA interactions; and to discover new potential biomarkers to provide new directions for the diagnosis, treatment, and prognosis of PCa.

(2) Proteomics–metabolomics

Proteomics allows the identification of a large number of proteins in biological samples, determining the structure and function of proteins, and studying protein modification and interaction networks. Metabolomics focuses on the study of metabolic end products and metabolic mechanisms by comparing the differences in metabolic changes between normal individuals and patients. Combining proteomics and metabolomics to analyze and study PCa helps people to fully understand the biological characteristics, protein regulatory networks, and metabolic abnormal cycles of PCa. Dougan et al. [128] analyzed the expression of the PXDN enzyme in PCa tissues using immunohistochemistry. They found that its expression was increased. Simultaneously, the metabolomics of the tissues was analyzed using LC-MS, revealing that PXDN promotes PCa progression by inhibiting oxidative stress, which leads to decreased apoptosis. PXDN may serve as a biomarker and potential therapeutic target for PCa. In another study, researchers analyzed PCa tissue proteomics using LC-MS, along with metabolomics analysis using MS, and proposed that amino acids such as lysine and arginine could play a role in PCa diagnosis as biomarkers, and that ceramides and sphingomyelins could help with prognosis [129]. Combined analysis of PCa tissues using proteomics–metabolomics can identify proteins or metabolites associated with PCa more precisely, which is a new direction for researchers to gain insight into the molecular mechanisms and pathological features of prostate tumors. Several findings in this area could provide new support for personalized treatment of patients.

(3) Transcriptomics–metabolomics

**Fig. 7.**
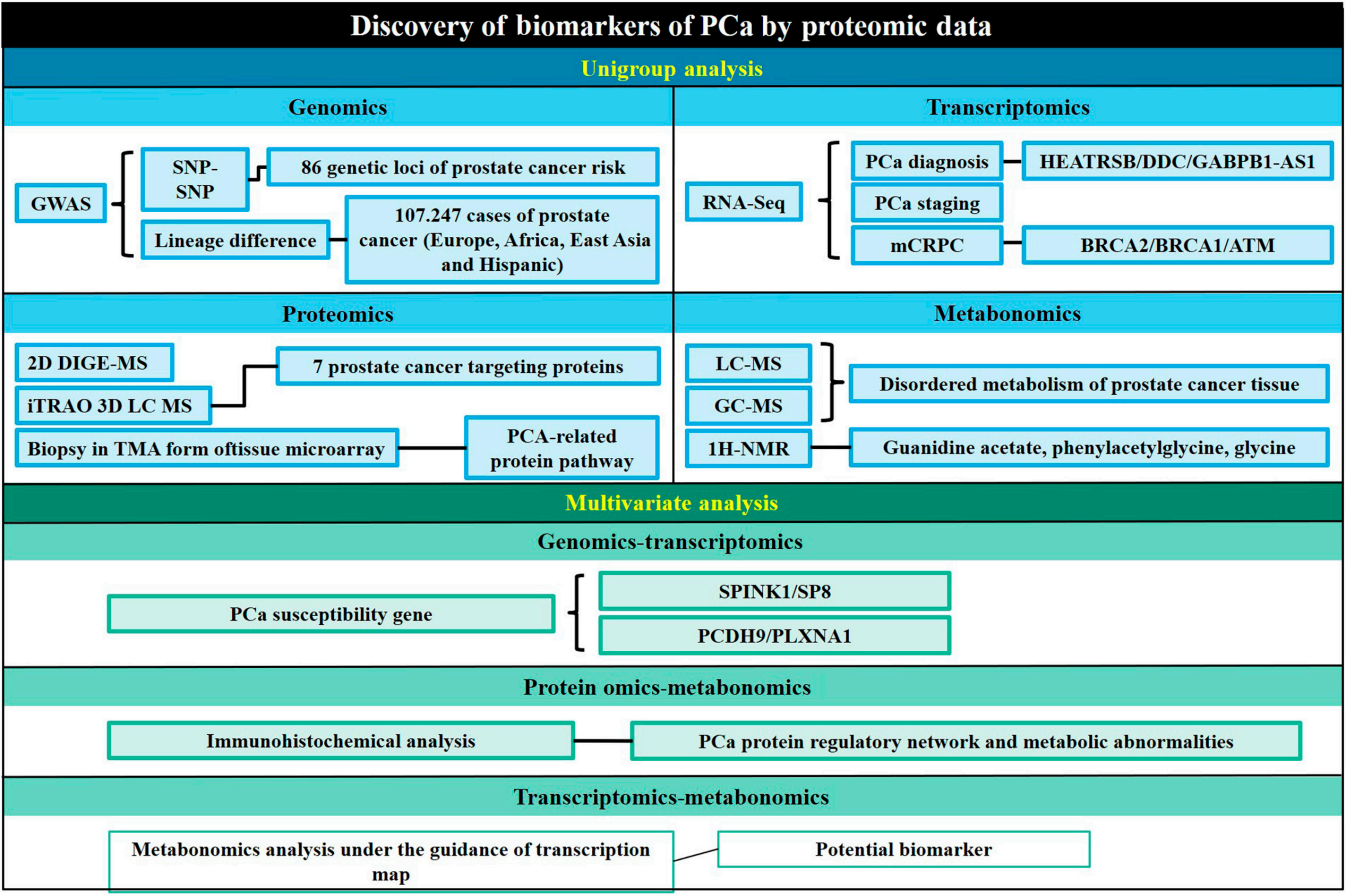
Summary dendrogram of methods used to discover new PCa biomarkers.

The combination of transcriptomics and metabolomics is a new and more cutting-edge approach to studying PCa. Through this approach, researchers can reveal the interactions between transcriptional regulation and metabolic pathways to discover potential biomarkers and therapeutic targets for PCa, helping to build a network of molecular mechanisms of the disease and advancing PCa research. In one study, researchers analyzed the NMR metabolic profiles of patients with different grades of PCa guided by transcriptional profiling and found that the levels of glucose, glycine, and 1-methyl nicotinamide of the patients showed significant changes in energy metabolism pathways [130]. Ren et al. [131] analyzed the patient’s PCa tissues and adjacent noncancerous tissues with transcriptomics and metabolomics, integrated analysis of PCa tissues and noncancerous tissues, proposed molecular interference mapping, identified multiple metabolic abnormal pathways, resolved the metabolic mechanism of PCa, and discovered multiple potential biomarkers. Shao et al. [132] used gas chromatography-mass spectrometry (GS-MS) and RNA-Seq to investigate the metabolic abnormalities in prostate tumors and adjacent normal sites at the molecular level to reveal the pathological mechanisms of abnormal metabolic pathways in PCa. Based on comprehensive molecular network analysis, it was found that the tricarboxylic acid cycle was hyperactivated in PCa tissues with elevated expression levels of branched-chain amino acid degradation genes and was closely related to the tricarboxylic acid cycle pathway. By combining transcriptomics and metabolomics, researchers can not only find the abnormal manifestations of PCa at the level of metabolite phenotype but also analyze the gene transcription mechanism of abnormal metabolism in-depth and obtain more accurate results from the comprehensive analysis of both metabolite production and gene expression, which can help to guide the diagnosis and treatment of PCa in the clinic (Fig. 7).

### Artificial intelligence analysis

With the continuous development of computer technology and the accumulation of big data patterns, artificial intelligence has become an efficient tool for solving complex problems, especially when facing massive data, as a new research method in scientific research. Machine learning is a commonly used technical tool in the field of artificial intelligence, which does not require explicit programming, and after building a model, the computer can automatically learn and improve the model according to the feedback of data and finally get the most optimized results. In PCa research, people can use all kinds of histological data to analyze PCa tissues or cells extensively. In the face of massive histological data, machine learning models can analyze their patterns, mine potential biomarkers, and contribute to personalized diagnosis and prognosis assessment for patients.

Machine learning can discover potential biomarkers from high-throughput biological data. In PCa research, potential biomarkers are mainly obtained by using data from genomics, transcriptomics, proteomics, and metabolomics to build appropriate machine learning models and analyze them. There have been many studies on deep learning of datasets based on genomics and transcriptomics to obtain genes related to pathological features and molecular mechanisms of PCa. Wu et al. [133] constructed gene expression prediction models for discovering potential causative genes for PCa, trained and analyzed the model’s performance using TCGA data, and finally found 137 genes that had a significant association with PCa, including 94 potential genes. The researchers also silenced 14 of these genes and found that the viability of cancer cells after gene silencing was significantly reduced, which further validated the accuracy of the machine learning model and proved that the machine learning method can locate genes associated with PCa with high accuracy. In machine learning, it is beneficial to analyze datasets using multiple features at the same time to produce more comprehensive results. In a study, researchers took advantage of the similarity of DNA molecular repair pathways between PCa and ovarian and breast cancer tumor cells to analyze multiple datasets by building a supervised deep learning model, and cross-cancer learning revealed that ADIRF, SLC2A5, C3orf86, and HSPA1B could be applied as biomarkers in the clinical diagnosis of PCa [134]. In clinical diagnosis, the introduction of machine learning methods can help the discovery of PCa biomarkers, which is important for both patients and healthcare organizations. Singireddy et al. [135] addressed the high resolution of RNA-Seq and combined it with machine learning techniques to discover the most discriminative transcripts for different stages of PCa, and used the above transcripts to further build a classification model, which can differentiate the stage of PCa progression. Liquid biopsy refers to the method of detection by analyzing the substances in body fluid samples, which is noninvasive and more advantageous in clinical detection than traditional tissue biopsy. Many studies have pointed out that machine learning can, by analyzing samples in conjunction with liquid biopsies, delve into abnormal molecular sites in PCa. Penney et al. [136] found that abnormalities in 25 metabolites, including 3-phosphoglyceric acid, were positively correlated with the Gleason score and could distinguish between prostate tumors and normal prostate tissues. Similarly, in another study, researchers built a machine learning model based on six protein variables and five clinical variables, and through continuous optimization, the model could distinguish PCa from prostate hyperplasia [137]. In addition to serum, machine learning models can also be applied to urine samples and analysis, which provide information for PCa diagnosis. Chen et al. [138] analyzed urine samples from PCa patients by constructing a model using Raman spectroscopy and convolutional neural network algorithms. Through the detection and analysis of Raman spectroscopy and the simulation and validation of convolutional neural networks, they found multiple potential biomarkers and proved that this method can be used to diagnose PCa. The study by Prestagiacomo et al. [139] constructed machine learning algorithm models of urine secretions (mainly proteins) from patients with PCa and patients with BPH and, after cross-validation analysis, found that sema7A and SPARC performed well and accurately in the models, and can be applied as potential biomarkers for PCa in clinical urine biopsy PCa diagnosis.

Prognostic biomarkers for PCa can monitor and predict the disease progression of patients, help doctors assess the disease development trend and survival time expectation of PCa patients, develop personalized treatment plans for patients, and provide precision medicine. Machine learning can efficiently process large amounts of biological data to discover potential associations and patterns, and the incorporation of machine learning techniques into PCa prognostic studies can help discover potential prognostic biomarkers and can further construct predictive models to forecast the future progression of a patient’s disease. A recent study in 2023 has identified a new biomarker for PCa by using proteomics data to develop support vector machines and artificial neural network frameworks, and identified a biomarker consisting of 16 proteins that can predict the sensitivity to anticancer drugs with an accuracy of about 92% [140]. The prognosis of PCa patients in terms of good health and survival is an important data in prognostic assessment, which is of great significance for the selection of treatment options. How to analyze the prognostic level of patients from the perspective of biomarkers is an important part of clinical research in PCa, and machine learning as a novel research method can help in this regard. Samaržija et al. [141] found that the Gleason score has a close association with the prognostic survival of PCa by using the recursive partitioning method using the TCGA data. Increased expression levels of SERINIC3 and CSAD improved prognosis. Even after receiving tumor treatment, PCa may still recur. Relevant studies have shown that the recurrence of PCa is associated with many factors. The use of machine learning tools to uncover biomarkers related to cancer recurrence is important in the optimization of clinical treatments. A cutting-edge study identified many critical gene pathways associated with the biochemical recurrence of PCa by constructing a deep learning recurrence-sensitive model, and the model can provide early prediction of patients’ recurrence risk [142]. Tong et al. [143] evaluated prognostic biomarkers of PCa using a protein–protein interaction network and validated the screening process using univariate regression analysis, which ultimately identified genes highly associated with PCa prognostic factor constructs, including UBE2C and CCNB1. In addition, Qiao et al. [144] established a support vector machine model to analyze PCa genomic data and demonstrated that MYLK is a powerful biomarker for BCR in PCa. In summary, machine learning, as a novel research method, can process a large amount of complex biological data, discover potential prognostic biomarkers, construct predictive models, which is important for predicting patient survival and recurrence, and provide new and powerful support for the determination and optimization of personalized treatment plans.

## Research on the Application of Biomarkers in Targeted Therapy and Diagnostics

Currently, PSA combined with digital rectal examination is still the most used method for early PCa screening in hospitals. Although many biomarkers have been helpful in detecting PCa, PSA is still the most frequently used detection index in hospitals. Serum PSA levels and CTC-based PSA mRNA concentrations were linearly plotted. Only PSA gene detected samples were plotted in dark green, blue, and purple and used for Spearman correlation coefficient calculations [145]. However, PSA is not a specific PCa biomarker; other prostate inflammation can also lead to increased PSA levels. Many noncancer men have elevated PSA levels (false-positive results), while some men with normal PSA levels were later diagnosed with PCa (false adverse effects) [146]. Therefore, the wide application of the PSA test may increase unnecessary prostate biopsies and cause the problem of overdiagnosis [147]. PHI, 4Kscore, and other parameters are often used as supplementary means of PSA detection in clinics, among which fPSA has been proven to improve the accuracy of PSA detection and reduce unnecessary prostate biopsy and is widely used in early detection of PCa [148]. Although targeted therapy for PSA is not common, PSA is often used as an essential biomarker to detect PCa, which can assist in the formulation of treatment plans and prognosis evaluation.

Besides PSA, PSMA, a membrane protein highly expressed in PCa cells, has been widely used in targeted therapy and diagnosis of PCa. PSMA can be used as an auxiliary method to stratify the risk of PCa. The expression of PSMA in PCa with Gleason score grade 3 is low, while grade 4 is high. The sensitivity and specificity of PSMA in detecting PCa are 84.1% and 95.2%, respectively, which indicates that PSMA plays an essential role in the pre-diagnosis and stratification of PCa and can help doctors clear the tumor classification and formulate appropriate treatment plans [149]. PSMA targeted therapy has attracted much attention as an excellent PCa biomarker. PSMA targeted therapy includes radioligand therapy, antibody conjugate therapy, and cellular immunotherapy [150], among which ^177^Lu-labeled PSMA radioligand therapy (^177^Lu-PSMA RLT) has been proved to have antitumor activity and safety, which can prolong the overall survival and progression-free survival of patients [151]. Through PSMA targeting nanoparticles, docetaxel can be selectively delivered to PCa tumors, showing significant therapeutic effects [152]. In addition, PSMA is often used for targeted PCa imaging by PET/CT, and ^68^Ga-labeled PSMA-11 PET/CT can be used for tumor localization, treatment of response evaluation’s disease, and disease recurrence monitoring. The positive rate of ^68^Ga-PSMA-11 PET/CT was 53.6% in patients with PCa whose serum PSA level decreased after radical treatment, and it was related to the clinical stage of biochemical recurrence [153]. For patients with early biochemical recurrence after prostatectomy, the study showed that ^68^Ga-PSMA-11 PET/CT greatly influenced the follow-up treatment scheme of 19% of patients and particularly affected 30% of patients. This detection can improve the individualized treatment scheme selection of patients with biochemical recurrence after radical prostatectomy [154].

**Fig. 8.**
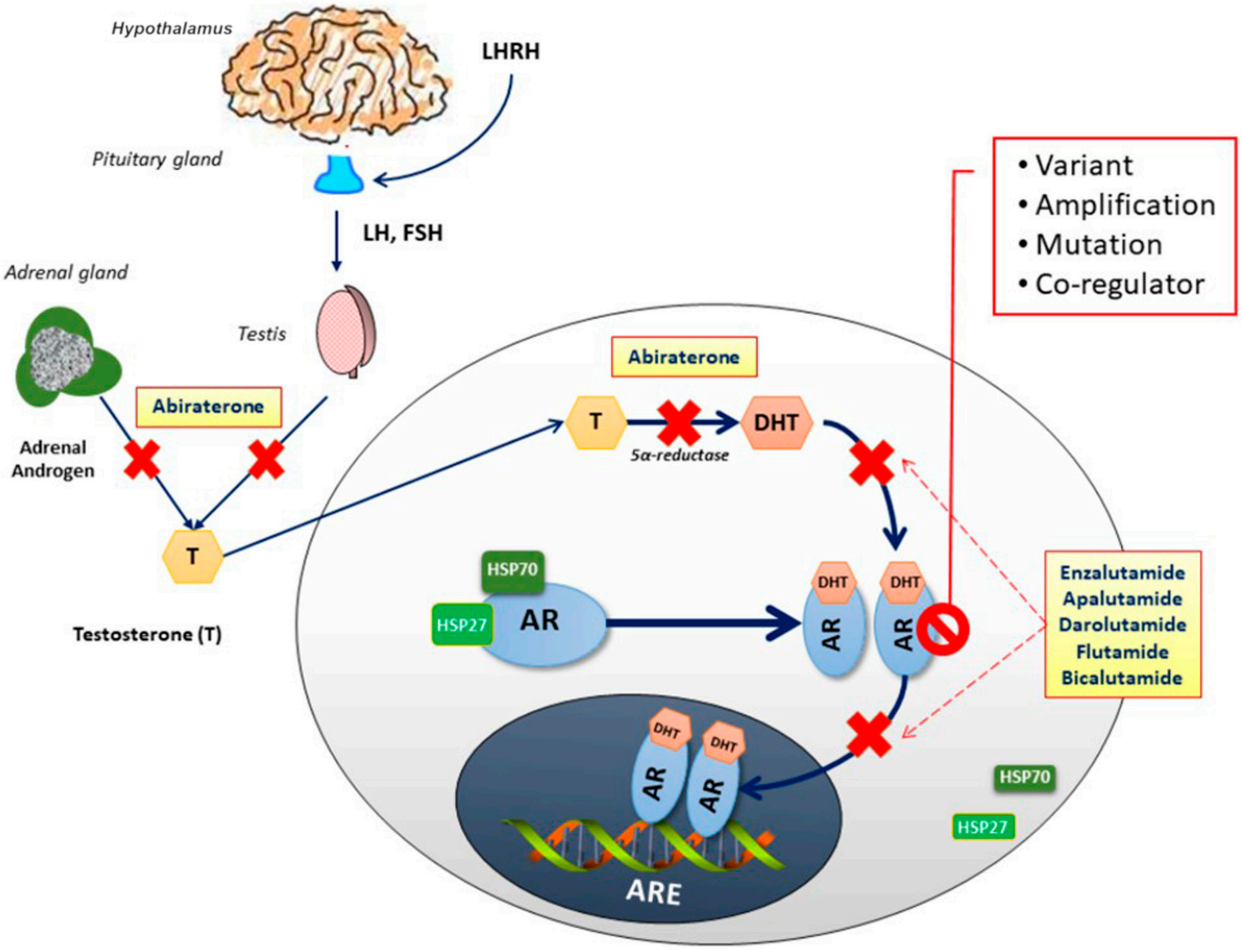
Androgen signaling through AR. T converted to active dihydrotestosterone (DHT) by 5α-reductase. Androgens bind AR, dissociating chaperones like heat shock proteins (HSPs). Ligand-bound AR homodimers translocate to the nucleus, bind androgen response elements (AREs), and regulate gene expression. Drugs: Abiraterone inhibits CYP17, blocking androgen synthesis; flutamide and bicalutamide reversibly and enzalutamide, apalutamide, and darolutamide irreversibly prevent androgen binding to AR [25]. Copyright 2021, *Cell.*

PSA and PSMA imaging are still the main biomarkers widely used in clinics, but AR, as a new biomarker, shows essential research and application prospects. Antiandrogen therapy can inhibit the proliferation of PCa cells by targeting AR, which is an essential basis for treating advanced PCa. In the last 10 years, various new hormone therapies have been applied in the treatment of PCa with castration sensitivity and metastatic castration resistance [25]. Their mechanisms of action are that abiraterone prevents androgen biosynthesis, and enzalutamide, apalutamide, and darolutamide inhibit AR translocation to the nucleus (Fig. 8) [25]. Studies have shown that lowering the testosterone level below 20 ng/dl can improve patients’ survival rate and delay the disease’s progress [155]. However, about one-third of patients showed primary resistance to AR-targeted drugs, and these patients were classified as castration-resistant prostate cancer (CRPC) [156]. The study also examined the response of CRPC to AR-targeted therapy. The splice variant of AR (AR-Vs) is considered a potentially important biomarker in PCa research [157]. Detecting the expression of AR-V7 in CTCs can affect the individualized treatment decision of CRPC [158]. Studies have shown that for patients with AR-V7-positive CRPC, taxol drugs can achieve a more prolonged overall survival than AR signaling pathway inhibitors. In contrast, for patients with AR-V7-negative CRPC, the application of AR signaling pathway inhibitors is better [159]. By detecting the status of AR-V7 to guide whether to choose AR signaling pathway inhibitors or paclitaxel drugs for treatment, the survival time of CRPC patients can be prolonged compared with the treatment decision without knowing the quality of AR-V7 [160].

**Fig. 9.**
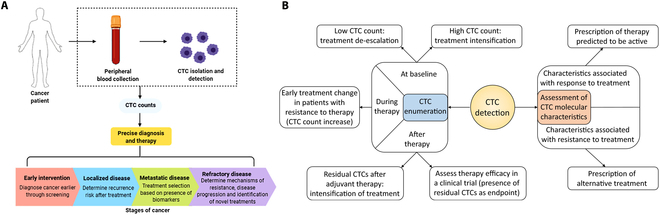
(A) The potential of CTCs as biomarkers for prognosis and outcomes at various stages of cancer [162]. Copyright 2021, *Mol Oncol.* (B) CTCs could improve the management of cancers in several ways [162]. Copyright 2021, *Mol Oncol.*

At the cellular level, CTCs have shown good diagnostic and therapeutic effect prediction value in PCa clinical trials (Fig. 9). CTC detection can be used for prognosis evaluation, treatment response monitoring, and disease progression judgment of PCa. They can also be used to evaluate tumor invasion and metastasis potential and acquired drug resistance to specific treatments. Although the clinical effect of CTC detection in PCa screening has yet to be verified, many studies have confirmed that the increase of CTC counts at baseline in metastatic PCa is related to poor prognosis. CTCs can be separated and enriched by antigen methods and detected by flow cytometry, which can obtain the prognostic value of PCa patients. CTC count is inversely related to the overall survival of patients, and the increase of CTC level often reflects the poor curative effect of the tumor on current treatment and the progress of patients [161]. In addition, CTC analysis can also evaluate the characteristics of patients’ genetic or acquired drug resistance to specific therapies, and guide the subsequent adjustment of precise treatment schemes [162]. At present, the CTC detection technology platform has developed rapidly. It has been partially commercialized, including the method of CTC enrichment and detection based on cell surface antigens or their physical properties. Among them, the application of microfluidic chip technology for CTC detection is considered a very promising research direction [163].

At present, PCa diagnostic methods, especially cancer cell identification, still have the problems of complicated detection operation and limited accuracy of detection results. In one study, researchers developed a molecular imaging method of multi-spectral deep ultraviolet (UV) microscopy, which uniquely identified basal cells, cavity cells, and inflammatory cells using the rich molecular information of unlabeled multi-spectral UV microscope. It can accurately and effectively identify benign and malignant glands, and has high fidelity without any staining procedures [164]. Terahertz wave is an electromagnetic wave with a frequency in the range of 0.1 to 10 THz, which has low ionization energy and is used for tissue imaging and spectral biomarker detection in the clinic. Terahertz spectroscopy is considered to be an effective tool for component analysis of tissue samples to identify cancer biomarkers, and it has great application potential [165].

**Fig. 10.**
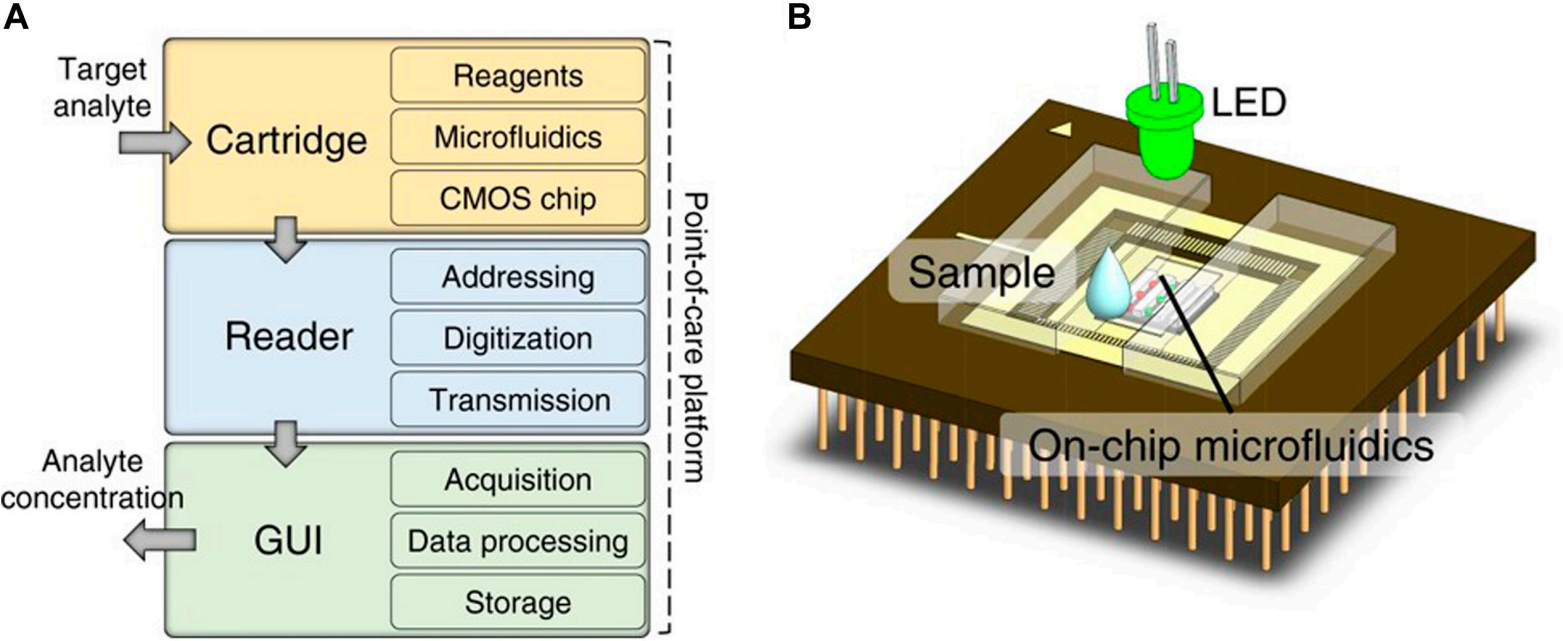
(A) Schematic structure of the microelectronic bedside metabolite biomarker measurement platform, showing a cartridge that requires only a drop of sample for measurement, a reader, and a computing device used as a graphical user interface [168]. Copyright 2021, *Microsyst. Nanoeng*. (B) Sketch of the multifunctional measurement cartridge device used in this work [168]. Copyright 2021, *Microsyst. Nanoeng*.

Conventional PCa diagnostic techniques, such as rectal examination, transrectal ultrasound, gray-scale ultrasound, and PET/CT, tend to suffer from certain shortcomings: Low-cost methods that are simple and easy to perform leave much to be desired in terms of sensitivity, whereas high-sensitivity techniques are usually more expensive [166]. As a result, researchers have begun to work on the development of smart analytical devices as a low-cost, high-sensitivity alternative for the detection of PCa. Several researchers have developed novel tools for immediate detection and bedside diagnostics: Uludag et al. [167] developed a novel integrated and fully automated microchip biosensor (MiSens) for the detection of PSA levels in serum. Annese et al. [168] reported on a microelectronic chemistry platform capable of measuring four metabolite biomarkers in human plasma in less than 2 min (Fig. 10). In addition to microchip sensors, nanomaterial-modified disposable screen-printed electrodes have also made significant progress in PCa detection, mostly for PSA detection [43], and only a few studies have reported the detection of PSMA [169,170].

In terms of optical biosensors, Jang et al. [171] constructed a PSA-α SPR biosensor for the detection of monoclonal antibodies in PCa cells, and Ertürk et al. [172] prepared a SPR biosensor for the detection of PSA in clinical samples by microcontact printing, which has the advantages of less inactivation of biorecognition molecules and a very small amount of samples required. In addition, although most of the intelligent analytical devices are based on blood detection, Khan et al. [173] proposed a paper-based graphene–polymer–gold biosensor, which can be used to detect PSA in saliva samples. The biosensor shows 94% concordance with the results of the enzyme-linked immunosorbent assay (ELISA) method. It is characterized by fast, high sensitivity, and high selectivity, with a storage period of approximately 7 weeks.

In addition to the above devices, artificial intelligence-assisted diagnosis, microfluidic chip integration, and other technologies also have broad application prospects in PCa detection. In summary, these emerging intelligent analytical devices are expected to make up for the shortcomings of the existing diagnostic methods and provide strong support for the accurate detection of PCa.

## Probe Application of PCa Biomarkers

In order to achieve high throughput and sensitivity detection of PCa biomarkers, the design and application of various specific binding probes have become a hot spot. These probes are mainly constructed according to molecular recognition principles such as antigen–antibody reaction and nucleic acid complementary pairing, and the target molecules are quantified or located by labeling means. It can be said that the discovery of PCa-related biomarkers provides an ideal target for the design of specific probes, and the targeted probes realize the efficient transformation and detection of these potential biomarkers. Also, there is a typical targeted recognition relationship between biomarkers and corresponding probes. This target and probe strategy not only enriches the technical means of accurate diagnosis of PCa but also provides the possibility for individualized treatment of the disease.

In the detection of PSA, the design of biological probe greatly affects the sensitivity and detection limit of the sensor. In order to obtain highly sensitive PSA sensors, researchers designed different types of biological probes, mainly including fluorescent probes and electrochemical probes. In the aspect of fluorescent probe, Jin et al. [161] designed a highly sensitive fluorescent probe using gold nanoparticles conjugated with dyes. Due to the high loading efficiency of dyes on the surface of gold nanoparticles and the strong fluorescence quenching effect of the dyes, the probe detected ultra-low concentration of PSA, with detection limit of 0.032 pg/ml [174]. In the aspect of electrochemical probes, Alnaimi et al. [175] constructed a label-free electrochemical biosensor by self-assembly of gold nanoparticles and DNA probes on a multi-walled carbon nanotube-modified electrode, and realized a lower sensitivity for PSA detection with a detection limit of 1 pg/ml. Biological probe is the key to realize ultra-sensitive detection of PSA. Whether it is fluorescent probe or electrochemical probe, using gold nanoparticles as probe carrier can significantly improve the detection sensitivity and reduce the detection limit of PSA. Soltani et al. [164] proves that high sensitivity, wide linear range, and low detection limit can be achieved by optimizing the design of biological probes. By adding anti-fluorescein Fab fragment–peroxidase conjugate and using multiple fluorescein-labeled hybridization auxiliary probes, the selective capture of PSA mRNA can be promoted. At the same time, the introduction of various oxidoreductases can further enhance the detection sensitivity [176]. At present, PSA detection probes with high sensitivity and specificity are still in great demand for early diagnosis and monitoring of PCa.

**Fig. 11.**
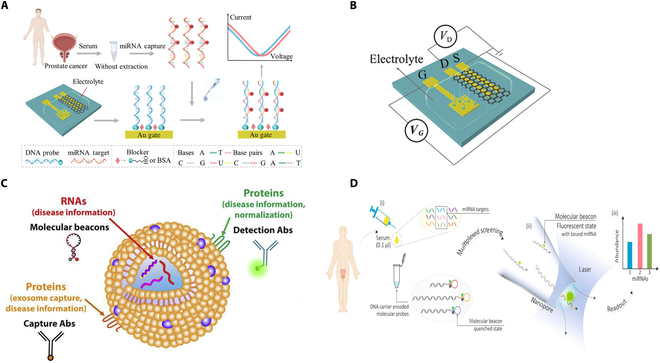
(A) Schematic diagram of unamplified detection of label-free miRNA-21 using the ssDNA-functionalized SGGTs biosensor [177]. Copyright 2023, *Adv. Sci.* (B) Configuration of the ssDNA-functionalized SGGT-based miRNA sensor [177]. Copyright 2023, *Adv. Sci*. (C) Configuration of the ssDNA-functionalized SGGT-based miRNA sensor [179]. Copyright 2019, *Biosens. Bioelectron*. (D) Workflow for detection of miRNAs directly from patient serum [180]. Copyright 2021, *Nat. Commun*.

Hitherto, the application of miRNA as a probe is still in the research stage. Research into the detection method of miRNA in body fluids or EVs can provide an evaluation of its accuracy and feasibility in the early diagnosis of PCa. Deng et al. [177] induced the Dirac voltage shift of designed graphene transistor (SGGT) transfer curve by hybridizing single-stranded DNA (ssDNA) probe with the molecular target of miRNA-21, and SGGT biosensor for ultra-sensitive and rapid quantitative detection of RNA-21 (Fig. 11A and B). In the clinical diagnosis and treatment of PCa, radiotherapy is one of the most common definitive treatment options, and miRNA as a probe is also of great significance. Studies have shown that miRNA, as an important regulator of cellular ionizing radiation (IR) reaction, is closely related to the radiosensitivity of many cancers. Liu et al. [174] performed miRNA probe hybridization chip analysis and found that miR-16-5p enhanced the radiosensitivity of PCa cells. This experiment may provide an alternative treatment for PCa [178]. In the process of using miRNA as a probe, EVs can provide high-precision disease information, especially when miRNA is combined with protein. Cho et al. [179] developed a method for simultaneous multiple in situ detection of EV miRNA and protein using nanomolecular beacons and fluorescent dye-coupled antibodies, and simultaneously detected EV miRNA and surface proteins in the captured EVs, with high specificity (Fig.11C). Cai et al. [180] developed a size-coded molecular probe for simultaneous electro-optic nanopore sensing of miRNA, allowing the direct detection of miRNA in serum to diagnose PCa (Fig. 11D). The development of miRNA probes can pave the way for the next generation of minimally invasive diagnosis and accompanying diagnostic tests of PCa.

TMPRSS2-ERG fusion gene event is one of the most common chromosome rearrangement events in PCa, and its probe has potential application value in the detection of PCa. Shrestha and others used hybridization technology to verify that TMPRSS2-ERG genome rearrangement occurred in prostate lesions, and confirmed that using gene rearrangement probes is a powerful tool for diagnosing PCa [181]. Gene rearrangement probes are also of great significance in the treatment of PCa. Zhou et al. [182] used inhibitors to inhibit the genome rearrangement of TMPRSS2-ERG, and proposed a new treatment strategy for PCa. In addition, Blackburn et al. [183] analyzed the data of genome rearrangement of various ethnic groups and found that the genome rearrangement of TMPRSS2-ERG is different due to different regions, which is expected to realize personalized diagnosis and treatment of PCa with this probe. With the in-depth understanding of biological functions and the continuous development of related detection methods, TMPRSS2-ERG gene rearrangement probes could become widely used in the clinical management of PCa and improve the diagnostic accuracy of PCa.

Sialic acid or glycosylation in glycoprotein modification plays an important role in the occurrence and progress of PCa. In patients with PCa, sialic acid in some glycosylated proteins has changed significantly, which provides potential application value as a probe for its diagnosis. Wen et al. [61] proposed that the study of sialylation in PCa using a new glucohormonal method could determine the potential biomarkers for diagnosis and therapeutic targets, and provide a new direction for sialylated glycoproteins as a probe.

CK as a probe has important clinical application value for the diagnosis of PCa. Liu et al. [184] used IL-6 as a probe to prepare a microfluidic bead-based protein extraction (BPE) array chip for multiple detection of cancer biomarkers. The detection limit is as low as 0.061 pg/ml, which has great application potential in clinical analysis. Kalra et al. [185] showed that CKs are an important factor in the progress of PCa, so adding CK antagonists may be an effective strategy for the new diagnosis and treatment of PCa. Using CKs as probes to study PCa, molecular transduction process can be tracked [186]. The diversity and specificity of CKs make them a potential diagnostic probe for PCa, which is expected to improve the accuracy of diagnosis and the implementation of individualized treatment, thus presenting a new perspective in the field of PCa diagnosis.

Radioactive drugs targeting PSMA are very important for the diagnosis, evaluation, and treatment of PCa. Radionuclide targeted molecular probes are helpful for accurate localization and treatment of lesions, especially for metastatic CRPC that is ineffective in conventional treatment. The molecular probes of PSMA include [^68^Ga]PSMA, [^18^F]PSMA, [Al^18^F]PSMA, [^99m^Tc]PSMA, and [^89^Zr]PSMA, which are widely used for diagnosis, and [^177^Lu]PSMA and [^225^AC]PSMA for treatment [187]. Gallium-68 is a positron radionuclide with a short half-life and is used for PET imaging. Generally, ^68^Ga-labeled PSMA PET has high sensitivity and specificity for primary and metastatic PCa lesions, which is superior to conventional imaging examination [188]. Besides radionuclide targeting molecular probes, there are also activatable fluorescent probes for targeting PSMA. Kawatani et al. [189] designed and synthesized PSMA glutamate carboxypeptidase (CP), which can activate a fluorescence detector. The probes can visualize the CP activity of PSMA in living cells and clinical specimens of PCa patients, and is expected to be used for rapid intraoperative detection and diagnosis of PCa.

Fibroblast activating protein (FAP), as a type II transmembrane serine protease, is extremely low in normal tissues but highly expressed in various solid tumors (including PCa), so it is widely studied as a tumor biomarker. Quinoline radionuclide probe based on FAP has been designed for imaging and targeted therapy, which can make up for the limitation of insufficient expression of PSMA in PCa, but its application scope may be limited to well-differentiated PCa. On the other hand, alkaline phosphatase (ALP), as an important tumor microenvironment enzyme marker, can significantly promote the progress of PCa. Yao et al. [190] developed a mitochondrial-targeted near-infrared activated fluorescence/photoacoustic (NIR FL/PA) probe, which realized efficient detection and localization of PCa-derived ALP, and could be used for aggregation-enhanced photothermal therapy. Generally speaking, continuously developing probes targeting multiple tumor markers such as FAP and ALP can not only make up for the limitations of single targeting but also realize individualized and precise treatment of PCa.

## Conclusion

The study of biomarkers for diagnosis and prognosis of PCa is poised for significant advancements driven by cutting-edge technologies. First, a single biomarker has some limitations, so the future development direction is to develop a multi-parameter joint detection system containing multiple biomarkers to improve the accuracy of diagnosis and prognosis judgment [191,192]. Second, the traditional tissue biopsy is traumatic, and the liquid biopsy technology can realize noninvasive monitoring by detecting blood or urine samples. Hence, the development of liquid biopsy technology is a significant development trend, but it still requires combining with the results of tissue biopsy to improve accuracy. In addition, using high-throughput proteomic detection platforms such as gene chips and protein chips can unravel new tumor biomarkers more comprehensively. The development of radionuclide imaging technology is also a promising direction to develop accurate medical imaging and treatment targeted by PSMA.

Moving forward, we need to focus on the following cutting-edge areas: (a) single-cell multi-omics sequencing technologies, which hold promise for uncovering novel biomarkers associated with cellular heterogeneity; (b) artificial intelligence and machine learning algorithms that will greatly enhance the modeling and predictive capabilities of multi-parametric biomarker models; (c) further optimization of liquid biopsy techniques, such as leveraging nanotechnology to improve detection sensitivity, or combining with other imaging modalities for precise localization; (d) integrating biomarker discovery with molecular imaging and targeted therapies to enable molecular-level precision diagnostics and personalized treatments.

Furthermore, prospective large-scale multicenter cohort studies are crucial for validating the clinical translational potential of newly discovered biomarkers. Another interesting prospect is the development of cell-free therapies using mesenchymal stem cell (MSC)-derived EVs. EVs can carry miRNAs, mRNAs, peptides, proteins, and CKs from MSCs to recipient cells, mediate intercellular communication, and promote the repair of damaged or diseased tissues and organs. Studies have shown that MSC EVs alone can realize the therapeutic potential of MSC. Therefore, the manipulation and application of MSC-derived EVs is expected to be a novel cell-free therapeutic modality for a wide range of diseases, such as cardiac, renal, hepatic, immune, and neurological disorders, as well as skin wound healing. Compared with live-cell delivery, MSC EVs are more stable and have reduced safety risks such as microvascular occlusion [193]. In addition, understanding the epidemiology and risk factors for some common surgical complications is essential to guide clinical management. For example, symptomatic urinary tract infection is a common complication after in situ neobladder urinary diversion, most often occurring in the first 3 months after surgery, with Pseudomonas aeruginosa as one of the main pathogens. Studies have shown that the risk of infection is not significantly affected by factors such as age and sex of the patient. Empirical treatment with amikacin antibiotics, which are effective against *Pseudomonas aeruginosa*, is recommended for these hospitalized patients [194]. Incorporating bioinformatics and artificial intelligence technology to establish an intelligent PCa diagnosis and early warning system and using big data to integrate and analyze massive detection information will also significantly improve the efficiency of diagnosis and prediction. Streamlining biomarker detection workflows and facilitating their efficient translation from the laboratory to the clinic will expedite the realization of precision medicine. Finally, the study of biomarkers needs the close cooperation of medicine, biology, laboratory medicine, computational biology, and other disciplines to obtain a formidable synergistic approach and emphasize the transformational application from laboratory to clinic. Interdisciplinary collaboration is vital for propelling advancements in this field and ensuring the rapid clinical implementation of novel biomarkers. This will enable newly discovered biomarkers to serve the clinic as soon as possible and genuinely realize the change from empirical medicine to precision medicine. Many newly discovered biomarkers cannot be used for the diagnosis and prognosis of PCa alone but usually need to be combined with other examination and evaluation methods, such as pathological biopsy and imaging (such as ultrasound, MRI, or CT scanning). The research on PCa biomarkers offers new opportunities, and interdisciplinary and transformational application is the future development direction.

The study of biomarkers necessitates close collaboration across disciplines including medicine, biology, experimental medicine, and computational biology to harness a formidable synergistic capability. Fundamental medical and biological research lays the theoretical groundwork for biomarker discovery. Clinical medicine provides invaluable pathological cases and clinical data. Experimental medicine is responsible for biomarker detection and evaluation, while computational biology contributes algorithmic support for high-throughput data analysis and biomarker integration modeling. The diverse disciplines play pivotal roles throughout the biomarker discovery, validation, and clinical translation pipeline. Consequently, strengthening interdisciplinary team collaboration is crucial for propelling advancements in this field.

Furthermore, we should strive to streamline biomarker detection workflows and facilitate their efficient translation from the laboratory to the clinic. This will enable newly discovered biomarkers to expeditiously serve clinical applications, genuinely realizing the transition from empirical medicine to precision medicine. Large-scale, multicenter prospective cohort studies are also a future priority to further substantiate the clinical translational value of biomarkers.
